# Treatments for rotator cuff calcific tendinitis: a systematic review and network meta-analysis of randomized-controlled trials

**DOI:** 10.1530/EOR-2024-0078

**Published:** 2025-06-30

**Authors:** Yuming Yao, Guang Yang, Shide Jiang, Bingzhou Ji, Hongfu Jin, Peiyuan Tang, Hengzhen Li, Bangbao Lu, Yusheng Li

**Affiliations:** ^1^ Department of Orthopedics, Xiangya Hospital, Central South University, Changsha, Hunan, China; ^2^ National Clinical Research Center for Geriatric Disorders, Xiangya Hospital, Central South University, Changsha, Hunan, China; ^3^ Department of Orthopedics, The Central Hospital of Yongzhou, Yongzhou, China

**Keywords:** calcific tendinitis, rotator cuff, non-surgical treatments, surgical treatments, randomized-controlled trials, network meta-analysis

## Abstract

**Purpose:**

- Various conservative and surgical treatments was clinically applied in rotator cuff calcific tendinitis (RCCT), the evaluation of distinction among all available intervention is still lacking. This study aims to systematically compare the efficacy and safety of these interventions and provide guidance for RCCT treatment.

**Methods:**

- The study utilized four electronic databases for literature retrieval and is registered in PROSPERO. Network meta-analyses (NMA) were conducted for continuous outcomes such as functional improvement and pain relief. For the outcome of calcification deposit resolution, due to discrepancies in statistical methods and insufficient data for pooling, a systematic review was conducted. The Cochrane Risk of Bias 2 tool was used to assess the risk of bias.

**Results:**

- A total of 33 randomized-controlled trials (RCTs) covering 26 treatment methods were included. In nonsurgical treatments, comprehensive physical therapy (PT) methods (ranked 1st in Surface Under the Cumulative Ranking (SUCRA)) showed the best performance in terms of functional improvement, followed by high-energy extracorporeal shock wave therapy (ESWT-H) + PT, which ranked 2nd. For pain relief, radial shock wave therapy (RSWT) + PT (ranked 1st) was most effective. In surgical treatments, arthroscopic bursectomy debridement of rotator cuff (ABD) + arthroscopic subacromial decompression (ASD) showed similar effects to ABD alone for functional improvement (SMD: −0.01, 95% CI: −0.54 to 0.57) and pain relief (SMD: −0.02, 95% CI: −0.51 to 0.43), with no significant differences observed. For calcification resolution, ultrasound-guided needling (UGN) + subacromial corticosteroid injection (SAI) demonstrated promising therapeutic potential.

**Conclusion:**

- Comprehensive PT demonstrates superior efficacy in improving functional outcomes, while RSWT + PT significantly alleviates pain. In terms of surgical interventions, ABD alone demonstrated similar clinical effects to ABD + ASD in both functional improvement and pain relief. However, there is currently no direct data to compare the effectiveness of operative versus nonoperative treatments for RCCT.

**Trial registration:**

- PROSPERO CRD42023476423.

## Introduction

Rotator cuff calcific tendinitis (RCCT) is a prevalent condition, manifesting in up to 20% of shoulders with pain symptoms and approximately 7.5% of asymptomatic shoulders (1). The supraspinatus tendon is most commonly affected most cases, followed by the infraspinatus tendon and the subscapularis tendon (2). Calcification can also occur simultaneously in several tendons, and the occurrence rate of involvement in the supraspinatus tendon and other tendons is approximately 90% (3). RCCT has a strong tendency to self-resolve and its natural course ranges from months to years (4), but this self-healing process can easily be hindered (5). Calcific tendonitis is clinically divided into three stages (6): pre-calcific, calcific and post-calcific. The pre-calcification stage is characterized by cellular changes at the site of calcification and fibrocartilage transformation; the calcification stage undergoes calcification formation, dormancy and resorption; the tendon at the site of calcification gradually heals and the fibers are rearranged after calcification and resorption (7). Due to the persistent presence of calcifications, besides causing shoulder pain and limited mobility, it can also lead to periarthritis of the shoulder, rotator cuff tears, osseous cystic changes and calcific tendinitis (8).

Various intervention approaches have been proposed and implemented over the years to manage RCCT. Conservative treatments such as physical therapy (PT) (9, 10) and nonsteroidal anti-inflammatory drugs (NSAIDs) (11) are first considered in the early stages of the disease; when the disease progresses to chronic calcific tendonitis, extracorporeal shockwave therapy (ESWT) (12, 13), focused shockwave therapy (FSWT) (14, 15), radial shock wave therapy (RSWT) (14, 16, 17), ultrasound therapy (US) (2, 18), ultrasound-guided needling (UGN) (19, 20, 21) and transcutaneous electrical nerve stimulation (TENS) (22) are considered. If nonoperative interventions are unsuccessful or undesired, surgical treatments can be utilized to treat the patient (23, 24). Although some studies have compared the clinical outcomes of various interventions, there has been no comprehensive analysis assessing the efficacy and safety of both surgical and nonsurgical treatments in managing RCCT.

Therefore, this study aims to conduct a network meta-analysis (NMA) to systematically evaluate and compare the efficacy and safety of various surgical and nonsurgical interventions for RCCT, addressing existing knowledge gaps. By synthesizing data from multiple studies, we hope to provide clinicians with robust evidence to support more informed decision-making in complex clinical scenarios. In addition, a deeper understanding of the relative strengths and limitations of each treatment modality will help optimize personalized care and ultimately improve patient outcomes in RCCT.

## Materials and methods

### Protocol and registration

This study followed the Preferred Reporting Items for Systematic Reviews and Network Meta-Analyses (PRISMA-NMA) (25) and was registered with the International Prospective Register of Systematic Reviews (PROSPERO: CRD42023476423).

### Literature search

A search was conducted in four electronic databases: MEDLINE (via PubMed), Web of Science, Embase and the Cochrane Library up to October 10, 2023. The following keywords were used: rotator cuff, rotator cuffs, subscapularis, infraspinatus, shoulder, calcinosis, calcific, tendinopathy, tendinitis, tendinosis, tendinoses, randomized-controlled trial (RCT), random, etc. In order to find as comprehensive a set of keywords as possible, the MeSH terms and EMTREE terms in PubMed and Embase, respectively, were scrutinized before the search. In addition, references that may be relevant to the treatment for RCCT were manually searched. The complete search strategies can be found in the Table S1 (see section on Supplementary materials given at the end of the article).

### Inclusion and exclusion criteria

Articles that met the following criteria were included in this systematic review and NMA: i) study patients confirmed and diagnosed with RCCT by radiographic or ultrasound examination; ii) evaluate at least two RCCT interventions; iii) at least one of the following outcomes was reported: improvement in function, pain relief or number of resolution of calcific deposits; iv) RCTs; and v) articles published in English. Studies were excluded if they met any of the following criteria: i) review articles, letters, meeting abstracts and expert opinions, ii) systematic review and meta-analysis, iii) animal experiments, and iv) studies that did not report qualifying outcome data or not provide a complete report.

### Study screening

After the automated removal of duplicates using the Endnote X9 software (X9, provided by University of Science and Technology of China, China), two authors first screened the titles and abstracts of retrieved literature for eligibility. Each potentially eligible study was then reviewed in full text to determine whether the inclusion criteria were met. If complete outcome data were not provided in the included RCTs, the reviewers contacted the authors of the trials to request additional information. If the authors did not respond, these RCTs were excluded from the systematic review and NMA. Disagreements between the two reviewers were resolved through discussion and negotiation, and if an agreement still could not be reached, a third senior author was requested to arbitrate the resolution.

### Data extraction

Before extracting information from the included studies, two authors created a standardized data extraction spreadsheet. These two authors then independently extracted the following data according to the spreadsheet: first author’s name, year of publication, first author’s country, intervention methods, post-intervention protocol, sample size (male/female ratio), mean age of patients, mean duration of symptoms, follow-up, complications, outcome measures and data of primary outcome measures. Interventions based on the same principles but different methods were categorized under the same treatment name (26). After data extraction, these two authors cross-checked the extracted information.

### Quality assessment

Two authors independently assessed the quality of included literature using the ROB-2 tool developed by the Cochrane Collaboration. This tool includes the following quality assessment items: i) randomization process, ii) deviations from intended interventions, iii) missing outcome data, iv) measurement of the outcome, and v) selection of the reported result. Based on the authors’ answers to the signaling questions, the risk of bias was assessed by categorizing the risk of each domain into three levels: ‘low risk of bias’, ‘some concerns’ and ‘high risk of bias’. If the risk of bias evaluation results of all areas is ‘low risk of bias’, then the overall risk of bias is ‘low risk of bias’; if the risk of bias evaluation results of some areas are ‘some concerns’ and there is no ‘high risk of bias’ area, then the overall risk of bias is ‘some concerns’; as long as the risk of bias evaluation results of one area is ‘high risk of bias’, then the overall risk of bias is ‘high risk of bias’ (27). In addition, each study within this network meta-analysis and systematic review underwent rigorous quality evaluation using the AMSTAR 2 (A Measurement Tool to Assess Systematic Reviews, Version 2), aimed at in-depth assessment of its quality. The AMSTAR 2 covers 16 key areas, including the comprehensiveness of literature search, the inclusion of risk of bias assessment and the appropriateness of meta-analysis methods, ensuring the evaluation’s comprehensiveness and accuracy (28).

### Statistical analysis

This systematic review and NMA utilized a Bayesian framework to integrate evidence from direct and indirect comparisons to assess the relative effectiveness of multiple treatment measures through the RJAGS and GEMTC packages in the R software (version 4.3.1). For continuous outcome data such as functional improvement and pain relief, effect sizes were expressed as the standard mean difference (SMD) and 95% confidence intervals (CI) (29); descriptive analyses were performed for the number of resolutions of calcific deposits. Differences between the two groups were considered statistically significant when the 95% CI of the SMD did not include zero. Markov chains were constructed by Markov chain Monte Carlo (MCMC) methods to generate sample sequences and thus estimate model parameters (30). Model fit and consistency were assessed using the deviance information criterion (DIC) and further validated by consistency versus inconsistency analysis (31). In addition, the degree of heterogeneity among studies was quantified using the *I*^2^ statistic in the consistency model within the RJAGS package, providing an estimate of the proportion of total variation in study estimates due to heterogeneity rather than chance. The effectiveness ranking of interventions was based on SUCRA values and ranking probabilities (32), which reflect the relative effectiveness of each intervention. To ensure the stability and convergence of the MCMC algorithm, the number of iterations was set to 20,000 and anneals to 50,000, using four MCMC chains with an initial value of 2.5 and a step size of 1. Convergence was assessed by trace and density plots to ensure the reliability of the parameter estimates. Publication bias was assessed by drawing funnel plots with regression lines using the Stata MP software (version 17).

Considering that patients undergoing surgical treatments typically have more severe conditions compared to those receiving nonsurgical treatments, with some considering surgery only after the failure of nonsurgical options (20), and since surgical treatments cannot be compared directly with nonsurgical treatments in NMA due to the lack of a connected evidence network, this systematic review and NMA categorized RCTs into two subgroups: surgical and nonsurgical studies. The analysis was then conducted separately for these two subgroups.

## Results

### Study selection

**Figure 1:**
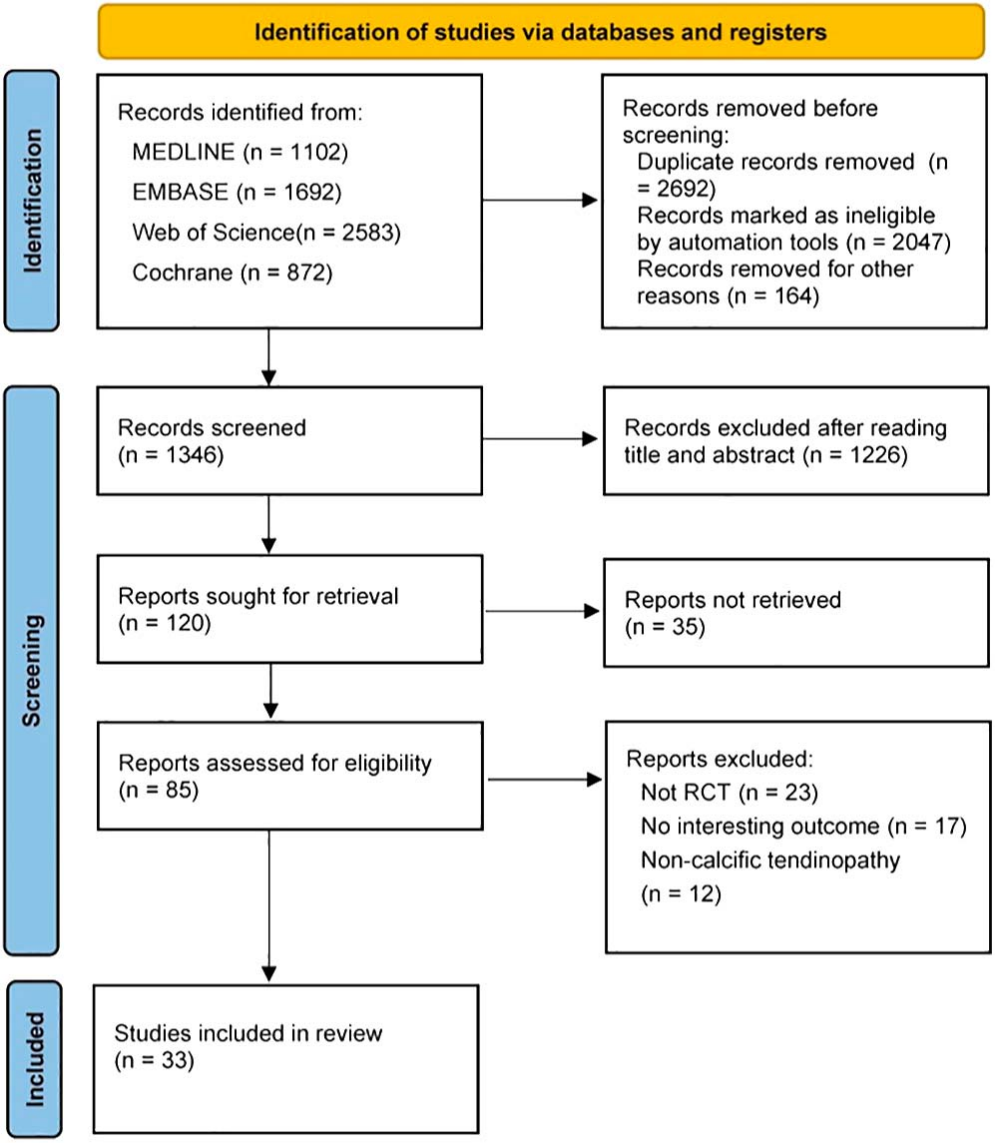
Flowchart of the study selection process.

A total of 6,249 studies were initially retrieved from the four electronic databases, and after the Endnote X9 software removed duplicates (*n* = 2,692), studies marked as ineligible by automation (*n* = 2,047) and for other reasons (*n* = 164), 1,346 studies remained. After screening titles and abstracts, 1,226 studies were excluded, and another 35 of the remaining 120 were excluded for not being retrieved. After reviewing the full text of the remaining 85 articles, 23 were excluded for not being RCTs, 17 for not having outcomes of interest to this study and 12 for non-calcific tendinitis. Ultimately, 33 articles met the criteria and were included in this systematic review and NMA. The specific search process is shown in Fig. 1.

### Study characteristics

The study included 33 RCTs published between 2003 and 2023, encompassing a total of 2,102 participants. Among them, 37.9% were male, with a median age of 52.2 years (range: 44.45–58.2 years). The mean duration of symptoms varied from 3 to 41.2 months, with a median last follow-up time after intervention of 8 months. Among the included studies, a total of 26 treatment modalities were reported, including four surgical and 22 nonsurgical interventions, but four of the nonsurgical interventions could not be included in the network analysis due to insufficient data. The specific baseline characteristics of the included studies are presented in Table 1.

**Table 1:** Baseline characteristics of included studies.

Study	Country	Interventions	Sample size (M/F)	Age, mean ± SD (y)	Mean symptom duration (mo)	Postprocedure protocol	FU (mo)	Outcome
Moosmayer *et al.* (35)	Norway	UGN+SAI	73 (22/51)	50.5 ± 8.5	32	PT, analgesics	24	OSS, DASH, VAS, EQ-5D-5L, resolution of deposit
		SAI	74 (27/47)	49.0 ± 8.8	32			
		PT	71 (25/46)	49.3 ± 9.0	34			
Verstraelen *et al.* (24)	Netherlands	ASD	20 (7/13)	52.7 ± 10.1	17.1	NA	6	VAS, CMS, DASH, ASES, radiological (radiographs, MRI, US)
		ABD	18 (3/15)	54.9 ± 7.7	20.3			
		ABD+ASD	24 (11/13)	53.2 ± 8.8	24.7			
Fatima *et al.* (12)	Pakistan	ESWT-H + PT	21	48.7 ± 6.74	>3	NA	3	NPRS, CMS, WORC, radiologic (US)
		PT	21	49.8 ± 7.54				
Kim *et al.* (14)	Korea	FSWT	20 (8/12)	52.80 ± 9.99		NA	0.25	VAS, CMS, ROM
		RSWT	20 (8/12)	52.70 ± 5.82				
Kuo *et al.* (17)	China	UGN	21 (8/13)	58.2 ± 7.9	13.8	Analgesics	3	VAS, CMS, SF-36, ROM
		RSWT	20 (8/12)	57.6 ± 9.4	21.3			
Al-Khair *et al.* (15)	Egypt	FSWT	15 (6/9)	50.53 ± 6.78	8.07	NA	3	VAS, ROM, SDQ, radiologic (US)
		RSWT	15 (5/10)	53.47 ± 10.04	8.93			
Louwerens *et al.* (13)	Netherlands	ESWT-H	41 (14/27)	51.6 ± 9.4	3.4	PT, analgesics	12	CMS, DASH, VAS, resolution of deposit, radiologic (radiographs)
		UGN+SAI	41 (15/26)	52.7 ± 8.7	3			
Duymaz & Sindel (16)	Turkey	RSWT+PT	40	54.33 ± 9.88	>12	NA	1	VAS, ROM, DASH
		PT	40	51.31 ± 8.86				
Darrieutort-Laffite *et al.* (33)	France	UGN+SAI	66 (17/49)	47.3 ± 9.2	30.8	NSAIDs/analgesics, no limitations of routine use of the shoulder	12	VAS, DASH, radiologic (radiographs)
		UGN+SSI	66 (26/40)	52.2 ± 9.5	34			
Papadopoulos *et al.* (44)	Greece	EAU+NSAIDs	20 (8/12)	46		Exercise protocol	18–24	VAS, ROM, IAOLDS, BDI, resolution of deposit
		NON	20 (9/11)	45				
Pieber *et al.* (42)	Austria		37 (14/23)	49.1 ± 10.6	>4	NA	120	Binder score, CMS, resolution of deposit, radiologic (radiographs)
		US						
		NON						
De Boer *et al.* (69)	Netherlands	UGN+SAI	11 (5/6)	53 ± 5.2	>6	Corticosteroid before treatment	12	NPRS, OSS, CMS, resolution of deposit, radiologic (radiographs)
		RSWT	14 (7/7)	53 ± 8.7				
de Witte *et al.* (40)	Netherlands	UGN+SAI	23 (11/12)	53.7 ± 7.3		NSAIDs/analgesics, ice second barbotage if persisting symptoms at 6 months	60	CMS, WORC, DASH, resolution of deposit, radiologic (US, radiographs)
		SAI	25 (12/13)	50.4 ± 7.2				
Battaglia *et al.* (39)	Italy	UGN+TA	20 (12/8)	46 ± 7	NA	Ice, NSAIDs, standardized exercise program	6	VAS, CMS, use of NSAIDs, resolution of deposit, radiologic (US)
		UGN+MA	20 (6/14)	51 ± 7				
Clement *et al.* (23)	UK	ABD+ASD	40 (9/31)	49.1 ± 9.0	6.75	Encouraged immediate movement	13	VAS, SF-12, DASH, CMS
		ABD	40 (12/28)	48.6 ± 10.3				
Kim *et al.* (19)	Korea	UGN+SAI	25 (2/23)	53.9 ± 7.75	>3	NSAIDs, no activity limitations	21.1	VAS, ASES, SST, resolution of deposit, radiologic (US, radiographs)
		ESWT-H	29(3/26)	57.4 ± 7.75			25.2	
Sabeti *et al.* (68)	Austria	ABD	10	46 ± 7.93	31.5	NA	9	CMS, VAS, resolution of deposit
		us-ABD	10	44.45 ± 7.99				
Kolk *et al.* (34)	Netherlands	RSWT	44 (12/32)	48 ± 9	24	Ice, use the arm normally	6	VAS, CMS, SST
		NON	38 (13/25)	46 ± 10.75	29			
de Witte *et al.* (48)	Netherlands	UGN+SAI	23 (11/12)	53.7 ± 7.3	>3	NSAIDs, ice, PT	12	CMS, WORC, DASH, VAS, resolution of deposit, radiologic (US, radiographs)
		SAI	25 (12/13)	50.4 ± 7.2				
Ioppolo *et al.* (45)	Italy	ESWT-M	23 (8/15)	57.09 ± 16.04	>4–6	NSAIDs before treatment	12	CMS, VAS, resolution of deposit, radiologic (radiographs)
		ESWT-L	23 (7/16)	51.65 ± 12.23				
Tornese *et al.* (70)	Italy		35 (14/21)	52.6	NA	PT, no analgesics	3	CMS, resolution of deposit, radiologic (radiographs)
		NON	17	53 ± 9.2				
		ESWT-HIR	18	52.2 ± 10.8				
Zhu *et al.* (21)	China	UGN-a	41 (26/15)	52.3 ± 6.75	11	NSAIDs, PT	9	VAS, shoulder function and satisfaction, radiologic (US, plain radiographs)
		UGN	40 (24/16)	53.1 ± 7.25	10			
Hsu *et al.* (71)	China	ESWT-H	33 (15/18)	54.4 ± 10	12.3	Lidocaine before treatment	12	CMS, VAS, resolution of deposit, radiologic (radiographs)
		NON	13 (4/9)	57.8 ± 9.5	11.1			
Albert *et al.* (37)	France	ESWT-H	40 (9/31)	46.6 ± 8.25	41.2	NSAIDs, no activity limitations	3	CMS, VAS, resolution of deposit, radiologic (radiographs)
		ESWT-L	40 (10/30)	47.5 ± 9.25	36.4			
Sabeti *et al.* (36)	Austria	ESWT-L	21	49.38 ± 8.37	>6	Subacromial infiltrations and PT were not allowed	3	CMS, VAS, radiologic (radiographs)
		ESWT-M	23	53.57 ± 8.80				
Cacchio *et al.* (47)	Italy	RSWT	45 (27/18)	56.12 ± 1.98	14	NA	6	UCLA, VAS, resolution of deposit, radiologic (US, MRI)
		NON	45 (28/17)	56.42 ± 2.09	13			
Sabeti-Aschraf *et al.* (46)	Austria	ESWT-feedback	25 (10/15)	52.96 ± 8.77	>6	NA	3	CMS, VAS, resolution of deposit, radiologic (CT, radiographs)
		ESWT-navigation	25 (12/13)	52.4 ± 7.74				
Krasny *et al.* (72)	Austria	UGN+ESWT-H	40 (24/16)	47.3 ± 8.7	36.3	NSAIDs, ice	4.1	CMS, resolution of deposit, radiologic (US, plain radiographs, MRI)
		ESWT-H	40 (15/25)	49.4 ± 7.8	30.5			
Pleiner *et al.* (43)	Austria	ESWT-H	23 (8/15)	54 ± 11	>6	Not standardized	7	CMS, VAS, resolution of deposit, radiologic (radiographs)
		ESWT-L	20 (4/16)	50 ± 8				
Gerdesmeyer *et al.* (49)	Germany	ESWT-H	48 (13/35)	51.6 ± 8.5	>6	PT, NSAIDs/ analgesics	12	CMS, VAS, resolution of deposit, radiologic (radiographs)
		ESWT-L	48 (16/32)	47.3 ± 8.5				
Pan *et al.* (22)	China	ESWT-H	32 (12/20)	55.21 ± 2.01	24.55	NA	3	CMS, VAS, manual muscle test, radiologic (US)
		TENS	28 (9/19)	58.00 ± 1.83	23.9			
Cosentino *et al.* (73)	Italy		70 (27/43)	51.8 ± 8.25		NA		CMS, resolution of deposit, radiologic (radiographs)
		ESWT-H	35 (15/20)		15		6	
		NON	35 (12/23)		14.5			
Perlick *et al.* (41)	Germany		80 (36/44)	48.4 ± 6.5	>12	NA	12	CMS, resolution of deposit, radiologic (US, plain radiographs, MRI)
		ESWT-H	40					
		ESWT-L	40					

ABD, arthroscopic bursectomy debridement of rotator cuff; ASD, arthroscopic subacromial decompression; ASES, American Shoulder and Elbow Society; BDI, Beck Depression Inventory; CMS, Constant Murley Score; CT, computed tomography; DASH, Disabilities of the Arm, Shoulder, and Hand questionnaire; EQ-5D-5L, EuroQol-5D-5L; ESWT, extracorporeal shock wave therapy; ESET-H, high-energy extracorporeal shock wave therapy; ESWT-HIR, ESWT-hyperextended internal rotation technique; ESWT-L, low-energy extracorporeal shock wave therapy; ESWT-M, middle-energy extracorporeal shock wave therapy; FSWT, focused shock wave therapy; IAOLDS, Instrumental Activities of Daily Living Scale; MA, methylprednisolone acetate; M/F, male/female; mo, month; MRI, magnetic resonance imaging; NA, not available; NON, sham/placebo; NPRS, numeric pain rating scale; NSAIDs, nonsteroidal anti-inflammatory drugs; OSS, Oxford Shoulder Score; PT, routine physical therapy; ROM, range of motion; RSWT, radial shock wave therapy; SAI, subacromial corticosteroid injection; SD, standard deviation; SDQ, shoulder disability questioner; SF-12, 12-Item Short Form Health Survey; SF-36, 36-Item Short-Form Health Survey; SSI, subacromial saline injection; SST, Simple Shoulder Test; TA, triamcinolone acetonide; TENS, transcutaneous electric nerve stimulation; UCLA, University of California at Los Angeles shoulder rating scale; UGN, ultrasound-guided needling ; UGN-A, UGN-with aspiration; US, ultrasound therapy; us-ABD, ABD of ultrasound positioning; VAS, Visual Analog Scale; WORC, Western Ontario rotator cuff index.

### Quality assessment

**Figure 2:**
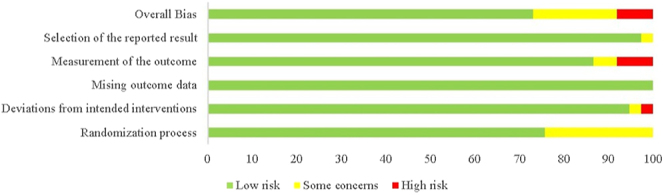
Cochrane risk of bias of included RCTs.

Figure 2 presents the risk of bias in the included RCTs. Although all studies mentioned ‘randomization’, only 25 provided details and adequately concealed the allocation process. Among these studies, five employed a double-blind design to enhance reliability and objectivity (23, 33, 34, 35, 36). Only 18 studies could effectively implement blinding due to the diversity of treatments and unique approaches (e.g. ESWT vs UGN). Two studies explicitly stated that blinding was not applied to outcome assessors (37, 38). Ultimately, six studies were judged to have some concerns (16, 21, 22, 24, 39, 40), three were at high risk of bias (41, 42, 43) and 24 were at low risk. Comprehensive assessment of bias risk is recorded in Table S2. The AMSTAR 2 assessment indicates that this network meta-analysis and systematic review demonstrated high quality and reliability in key areas.

**Figure 3:**
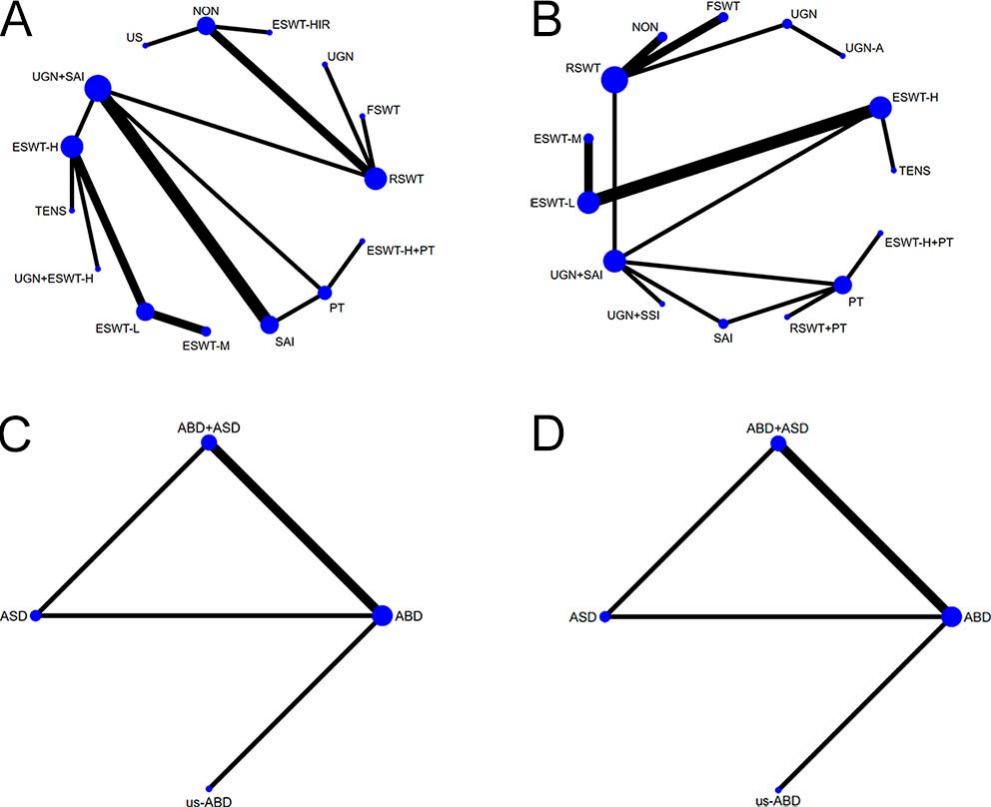
(A) Network 1: nonsurgical treatments (function score). (B) Network 2: nonsurgical treatments (pain score). (C) Network 3: surgical treatments (function score). (D) Network 4: surgical treatments (pain score). Note: within this network diagram, each intervention is represented by a node, the size of which reflects the number of patients included, and the thickness of the lines indicates the number of studies included – the larger the node, the more patients covered, and the thicker the line, the more studies pertaining to that treatment. ABD, arthroscopic bursectomy debridement of rotator cuff; ASD, arthroscopic subacromial decompression; ESWT, extracorporeal shock wave therapy; ESET-H, high-energy extracorporeal shock wave therapy; ESWT-HIR, ESWT–hyperextended internal rotation technique; ESWT-L, low-energy extracorporeal shock wave therapy; ESWT-M, middle-energy extracorporeal shock wave therapy; FSWT, focused shock wave therapy; NON, sham/placebo; PT, physical therapy; RSWT, radial shock wave therapy; SAI, subacromial corticosteroid injection; SSI, subacromial saline injection; TENS, transcutaneous electric nerve stimulation; UGN, ultrasound-guided needling; UGN-A, UGN-with aspiration; US, ultrasound therapy; us-ABD, ABD of ultrasound positioning.

### Intervention effect comparison

In the evaluation of functional improvement with nonsurgical treatments, a total of 15 therapeutic methods were compared. Among these comparisons, 11 groups showed significant differences. The comparison between UGN+SAI and SAI was the most common, with three studies; followed by the comparisons between RSWT and NON, and between ESWT-H and ESWT-L, each with two studies. The analysis revealed that, in terms of functional improvement, ESWT-H+PT was significantly more effective than SAI (SMD: 11.24, 95% CI: 0.79–21.77), and PT was also significantly more effective than ESWT-H (SMD: 11.05, 95% CI: 0.46–21.52) (Table S3).

Fifteen different treatment modalities were compared in the assessment of pain relief with nonsurgical treatments. The comparison of therapeutic efficacy between ESWT-H and ESWT-L was explored in three studies, but no significant differences were found. No statistical differences were observed in the evaluation of functional improvement and pain relief with surgical treatments, even though four different methods were compared (Fig. 3; Tables S4, S5, S6).

**Figure 4:**
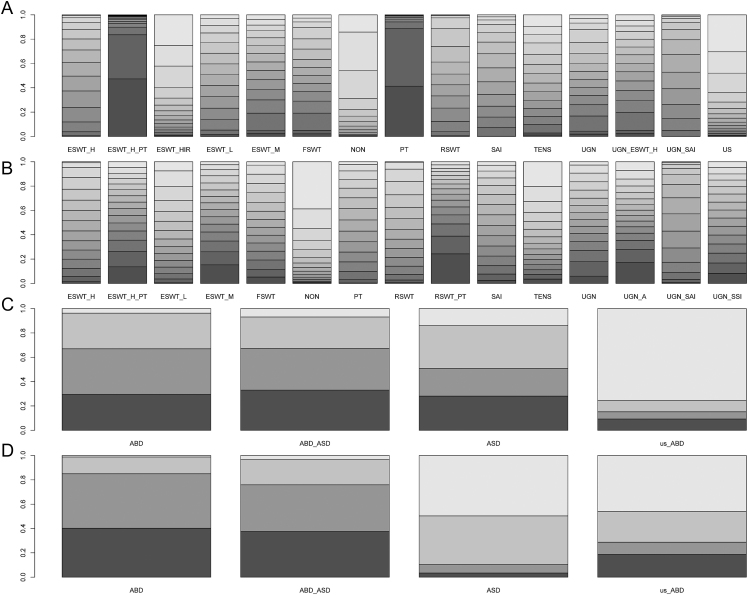
(A) Cumulative probability ranking for nonsurgical treatments (function score). (B) Cumulative probability ranking for nonsurgical treatments (pain score). (C) Cumulative probability ranking for surgical treatments (function score). (D) Cumulative probability ranking for surgical treatments (pain score). ABD, arthroscopic bursectomy debridement of rotator cuff; ASD, arthroscopic subacromial decompression; ESWT, extracorporeal shock wave therapy; ESET-H, high-energy extracorporeal shock wave therapy; ESWT-HIR, ESWT–hyperextended internal rotation technique; ESWT-L, low-energy extracorporeal shock wave therapy; ESWT-M, middle-energy extracorporeal shock wave therapy; FSWT, focused shock wave therapy; NON, sham/placebo; PT, physical therapy; RSWT, radial shock wave therapy; SAI, subacromial corticosteroid injection; SSI, subacromial saline injection; TENS, transcutaneous electric nerve stimulation; UGN, ultrasound-guided needling; UGN-A, UGN-with aspiration; US, ultrasound therapy; us-ABD, ABD of ultrasound positioning.

In addition, in the NMA, we ranked the effects of nonsurgical and surgical treatments on functional improvement and pain relief based on SUCRA values (see Table S7). The results showed that, among nonsurgical treatments, ESWT-H+PT had the best performance in functional improvement (SUCRA ranked second) and ranked third in pain relief. PT alone had the highest ranking in functional improvement (SUCRA ranked first), but there was no significant difference compared with ESWT-H+PT (SMD: −0.06, 95% CI: −8.45 to 8.22). In pain relief, RSWT+PT had the best effect (SUCRA ranked first). In surgical treatments, ABD+ASD and ABD showed significant advantages in both functional improvement and pain relief (both SUCRA ranked first; Fig. 4).

### The failure of nonsurgical treatment

Five studies reported on patients who underwent surgical treatment after failure or ineffectiveness of nonsurgical treatment. Seven out of 41 patients (7/41) underwent arthroscopic subacromial bursa resection combined with intraoperative puncture therapy (13). Papadopoulos and colleagues noted that one patient out of 20 (1/20) in the control group underwent surgical intervention following unsuccessful conservative pharmacotherapy (44). Moreover, among the 66 patients treated with SAI, 16 (16/66) ultimately required surgical intervention for the shoulder (33). In a RCT comparing UGN+SAI to SAI alone, four patients out of 48 (4/48) received surgical treatment following the intervention (40). Furthermore, one patient in the UGN group ultimately underwent subacromial debridement and decompression surgery due to intractable pain (40).

### Complications

A total of 21 studies reported the occurrence of complications or adverse events, with six studies (17, 21, 34, 36, 45, 46), explicitly noting no side effects or complications. Most reported adverse events were mild and transient, predominantly manifesting as systemic reactions including temporary dizziness, nausea, headache and vasovagal responses. Localized reactions were primarily characterized by injection site hematoma, exacerbated pain and superficial skin manifestations such as petechiae, minor bruises, bleeding and erythema. Shoulder-specific complications included subacromial bursitis, postoperative adhesive capsulitis and frozen shoulder syndrome. In addition, rare adverse events such as hypertensive episodes and infections were documented. Notably, these complications typically resolved spontaneously within a short duration (ranging from minutes to hours) post-treatment and demonstrated no significant impact on patients’ long-term clinical outcomes.

### Post-treatment calcific deposits resolution

Of the 33 included RCTs, 24 provided data on the resolution of calcific deposits, with a median final follow-up of 5 months, ranging from 3 weeks to 12 months. The therapeutic approaches encompassed a variety of combinations, including UGN+SAI, ESWT-H, ESWT-M, RSWT and so on. At the final follow-up, patients undergoing UGN+SAI treatment exhibited an average complete resolution rate of 56.82% for calcific deposits; those receiving ESWT-M and ESWT-H had respective rates of 34.78 and 30.00%. Moreover, data on the average reduction in the size of calcific deposits were reported in five studies (19, 44, 47, 48, 49), indicating notable differences between them. Details on the resolution numbers and changes in the size of calcific deposits are presented in Table S8.

### Model diagnostics and consistency assessment

The study conducted consistency and inconsistency tests on four subgroups – nonsurgical treatment for functional improvement, nonsurgical treatment for pain relief, surgical treatment for functional improvement and surgical treatment for pain relief – finding that all subgroups’ DIC differences were less than 5 (40.10 vs 40.13, 39.98 vs 40.00, 9.11 vs 9.88, 7.66 vs 9.11, respectively), indicating good consistency across the subgroups (50). In addition, the heterogeneity within each subgroup was assessed using the *I*^2^ statistic, yielding results of 6, 5, 24 and 0%, respectively. To further assess model stability and the reliability of parameter estimates, trace plots and density plots were also generated. Detailed illustrations are presented in the Figs S1 and S2.

### Publication bias assessment

**Figure 5:**
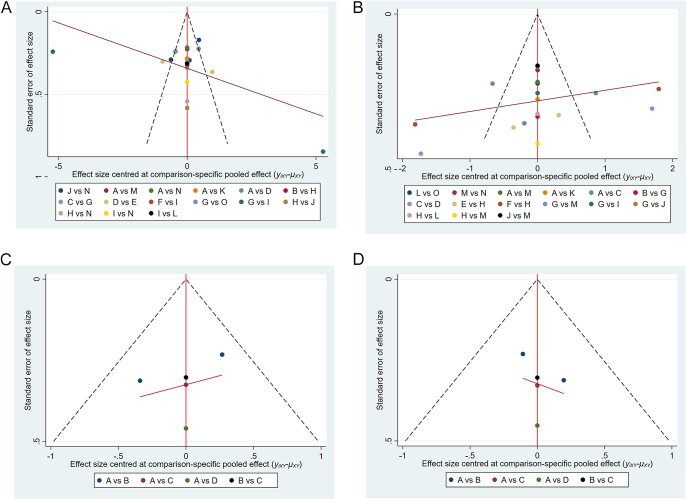
(A) Funnel plot of nonsurgical treatments (function score). (B) Funnel plot of nonsurgical treatments (pain score). (C) Funnel plot of surgical treatments (function score). (D) Funnel plot of surgical treatments (pain score). In (A), A=ESWT-H, B=ESWT-H+PT, C=ESWT-L, D=ESWT-M, E=FSWT, F=NON, G=PT, H=RSWT, I=RSWT-PT, J=SAI, K=TENS, L=UGN, M=UGN-A, N=UGN+SAI, O=UGN+SSI; in (B), A=ESWT-H,B=ESWT-HIR, C=ESWT-H+PT, D=ESWT-L, E=ESWT-M, F=FSWT, G=NON, H=PT, I=RSWT, J=SAI, K=TENS, L=UGN, M=UGN+ESWT-H, N=UGN+SAI, O=US; in (C) and (D), A=ABD, B=ABD+ASD, C=ASD, D=us-ABD.

Funnel plot analysis for the four subgroups demonstrated overall symmetry in the distribution of points, suggesting a low risk of publication bias within each subgroup. Although a few outliers were observed in certain subgroups, possibly indicating publication bias for individual study results or heterogeneity among studies, these did not appear to significantly distort the overall quality of evidence. The red regression line indicated the relationship between effect sizes and standard errors, which in most subgroups did not show a strong correlation, reinforcing the robustness of the overall evidence. For detailed illustrations, see Fig. 5.

## Discussion

This systematic review and NMA integrated 33 RCTs covering 26 intervention methods, aiming to evaluate the effects of these methods on functional improvement, pain relief or calcific deposit resolution in patients with RCCT. The results showed that up to the last follow-up time (median: 8 months), ESWT-H+PT appeared to be the most effective nonsurgical treatment for functional improvement (second in SUCRA) and pain relief (third in SUCRA). PT alone showed the best results in functional improvement (first in SUCRA), but there was no significant difference compared to ESWT-H+PT (SMD: −0.06, 95% CI: −8.45 to 8.22). For pain relief, RSWT+PT was superior (first in SUCRA). In addition, ESWT-M was more effective than both ESWT-H and ESWT-L in post-treatment functional improvement and pain relief. In surgical treatments, both ABD+ASD and ABD demonstrated superior outcomes in functional improvement and pain relief (each ranked first in SUCRA). Nevertheless, due to the limited number and quality of RCTs, only nonsurgical treatments for functional improvement showed statistically significant differences, and due to the lack of direct comparative studies between nonsurgical and surgical treatments, it is impossible to rank their effectiveness. Regarding the resolution of calcific deposits post-treatment, UGN+SAI appeared to be relatively more effective, with 56.82% of the calcific deposits completely resolved.

In the treatment of RCCT, both nonsurgical and surgical treatments have their respective indications. Nonsurgical treatment is usually suitable for patients with mild-to-moderate symptoms, which includes PT (such as hot (51) and cold compresses), ESWT, RSWT, FSWT, UGN, SAI, TENS, etc. The primary goals of these treatment methods are to alleviate pain, improve shoulder function and promote the natural absorption of calcifications (52). For instance, ESWT and RSWT have been clinically proven to effectively promote the absorption of calcifications and reduce pain (47, 49). UGN treatment involves precise localization and needling of the calcified area under ultrasound guidance to promote the decomposition and absorption of calcifications (17, 35). For patients who do not respond to nonsurgical treatment and have severe symptoms affecting daily activities, surgical treatment becomes a more suitable choice (24, 53). Surgical treatment methods include ABD, arthroscopic subacromial decompression (ASD) and the combined use of these two methods. ABD is primarily used for removing inflamed bursa and clearing calcifications, while ASD aims to reduce rotator cuff compression and improve the range of motion of the shoulder. These surgical methods can effectively relieve symptoms and provide a more fundamental solution for patients who respond poorly to nonsurgical treatment.

In the context of comparing nonsurgical treatments for chronic RCCT, a NMA conducted by Wu *et al.* in 2016 found that UGN, RSWT and high-energy FSWT had significant advantages in relieving pain and eliminating calcific deposits (52), consistent with the results of this study. However, there has not been a NMA comparing surgical treatments for RCCT. A recent systematic review and meta-analysis by Angileri *et al.* suggested that surgical treatment might be more effective than nonsurgical treatment in improving shoulder function and pain relief, although which specific surgical method is most effective remains unclear (54).

ESWT works by focusing high-amplitude sound waves on specific body parts, effectively dissolving calcifications, promoting tissue healing, reducing inflammation and alleviating chronic pain (16). ESWT encompasses a broad category of all external shock wave treatments and is generally classified into three levels based on energy density: low (<0.08–0.10 mJ/mm^2^), medium (0.08–0.28 mJ/mm^2^) and high (>0.29–0.60 mJ/mm^2^) (55). The treatment mechanism of ESWT primarily involves mechanical wave stimulation to decompose and absorb calcific substances and stimulate angiogenesis, thereby improving local blood circulation and tissue repair for treating calcific tendinitis (56). Studies show varying effectiveness of different energy densities of ESWT in improving function and relieving pain (57). Previous RCTs have compared ESWT-H with ESWT-L and ESWT-M with ESWT-L (37, 38, 43, 45, 49). However, there is a lack of direct comparisons between ESWT-M and ESWT-H in terms of functional improvement, pain relief and resolution of calcific deposits. The current evidence indicates that ESWT-M is more effective in improving symptoms and relieving pain in RCCT, and seems to have an advantage over ESWT-H in resolving calcific deposits post-treatment. A possible explanation for the superiority of ESWT-M over ESWT-H could be that medium energy levels offer a balanced treatment approach, potentially more effective in stimulating biological processes such as angiogenesis or cell proliferation, thereby leading to better outcomes in pain relief and functional improvement. Moreover, ESWT-M, with its moderate energy output, may reduce the risks of tissue damage or side effects associated with high-energy treatments. However, these conclusions need further validation and exploration in future studies due to the lack of direct comparative research between ESWT-M and ESWT-H.

FSWT and RSWT are two subtypes of ESWT, differing in the transmission of shock waves (52). FSWT uses focused shock waves for precise action on deeper tissues, while RSWT emits radial waves that cover a broader area, typically used for superficial tissue treatment (58). For example, a study on treating heel spurs found that both RSWT and FSWT significantly reduced pain, but RSWT was superior in terms of pain reduction and duration (59). This aligns with findings from this study that nonsurgical treatment combining RSWT and PT is most effective in pain relief. Traditional PT methods for RCCT usually include moderate rest, cold compresses to reduce inflammation and pain, hot compresses to promote blood circulation, electrotherapy for pain relief, ultrasound treatment, iontophoresis with acetic acid, manual therapy and stretching and strengthening exercises to enhance joint mobility and muscle strength (60, 61). In this study, PT proved superior in improving function and relieving pain compared to TENS and therapeutic ultrasound, possibly due to the combination of various PT methods used in some studies (12, 16, 35).

UGN, also known as lavage, barbotage or irrigation (62), is a minimally invasive treatment method primarily involving needle-based washing, with or without suction. UGN is often used in combination with SAI, a therapy increasingly valued by clinicians for its enhanced effectiveness and precision. Clinically, UGNs advantage lies in its ability to precisely locate calcific deposits under ultrasound guidance, providing targeted treatment (13). Compared to SAI alone, UGN combined with SAI shows more significant results in reducing inflammation and promoting the absorption of calcific materials. Studies indicate that this combined therapy not only alleviates pain but also improves joint function and enhances patients’ quality of life (63). In this study, UGN+SAI ranked third in both functional improvement and pain relief, further confirming the conclusions of previous research.

In clinical practice, surgical methods are often used to treat the late stages of chronic RCCT, aiming to improve patients’ pain and functional status. Common surgical procedures include ASD and ABD, which focus on removing inflammatory tissue and promoting healing of the rotator cuff. Although ASD is considered necessary in certain cases, particularly in the presence of anterolateral impingement, our study did not demonstrate any additional clinical benefit from ASD. Unlike the common practice of some surgeons who routinely combine ASD with ABD, our NMA revealed no significant difference in functional improvement or pain relief between ABD + ASD and ABD alone. This finding may be related to the characteristics of the study population, which primarily consisted of patients with RCCT, most of whom did not exhibit clear signs of subacromial impingement. While ASD may effectively relieve rotator cuff compression in patients with acromial anatomical abnormalities, its additional benefits appear limited in those without such abnormalities. Therefore, our results suggest that ASD is not necessary for all patients with RCCT, unless there are clear indications such as evident subacromial impingement symptoms. Further high-quality RCTs are needed to confirm these findings, particularly in patients with concomitant subacromial impingement.

It is noteworthy that the failure rate of nonsurgical treatment should also be an important consideration for clinicians when selecting RCCT interventions. Among the included studies, although only five reported patients who required surgery after the failure or ineffectiveness of nonsurgical treatment, the failure rates are indeed concerning. Specifically, in the ESWT-H group described by Louwerens *et al.* (13), seven out of 41 patients (7/41) eventually required arthroscopic debridement and intraoperative needling, resulting in a failure rate of 17%. In another study (33), 16 out of 66 patients treated with UGN+SAI eventually underwent surgery, yielding a failure rate of 24%. Patients who undergo surgery after the failure of nonsurgical treatment face increased medical costs and prolonged disease duration, which clinicians should aim to avoid in their decision-making. Therefore, conducting further relevant RCTs to clarify the failure rate of nonsurgical treatment and identify factors influencing it is essential (64, 65, 66, 67).

The removal of calcific deposits is widely regarded as a key outcome in the surgical treatment of RCCT, particularly during arthroscopic procedures aimed at removing calcifications. Although clearing calcific deposits is crucial for alleviating symptoms and improving shoulder function, RCT data on this outcome are relatively limited, especially in studies directly comparing surgical and nonsurgical treatments. Most existing studies focus on short-term clinical outcomes, while there remains insufficient evidence to support the role of calcific deposit removal as a predictor of long-term postoperative success. In our review, the study by Sabeti *et al.* highlighted the importance of calcific deposit removal during arthroscopic surgery (68), particularly in the comparison between standard localization and ultrasound-guided localization techniques. Their findings demonstrated a significant increase in the rate of calcific deposit removal following surgery, with ultrasound-guided localization markedly enhancing the detection and removal efficiency of the calcifications.

## Strengths and limitations

First, this is the first NMA that comprehensively compares various surgical and nonsurgical treatment methods for RCCT. Compared to previous network meta-analyses, it not only includes a broader range of nonsurgical treatments but also considers surgical treatments for the first time, offering a more comprehensive perspective on the overall management of RCCT. Second, all included studies are RCTs, providing a series of high-quality evidence for clinical practice and effectively reducing the risk of selection bias. In addition, this analysis compares the relative benefits of treatment methods, regardless of whether they have been directly compared, offering valuable references for doctors and patients. Finally, by using quantitative methods to assess the effectiveness and certainty of various treatments, the transparency of treatment effect comparisons is enhanced, guiding future research directions.

However, this study also has some limitations. First, although all included studies are RCTs, there may be differences in the quality of their design, execution and reporting, leading to heterogeneity in the study results. Second, while no significant inconsistencies were found, the single closed loop in each network diagram – caused by three-arm RCTs – might limit the robustness of indirect comparison analyses. Third, due to the lack of RCT directly compared surgical and nonsurgical treatments, the clinical effects distinction among those intervention has not yet been estimated. Moreover, considering the rapid development of medical techniques and methods, the included studies might not cover the latest treatment options, potentially affecting the practical applicability of the conclusions. Future research needs to include more independent multi-arm studies to enhance the credibility and interpretability of network meta-analyses.

## Clinical implications

This study offers evidence-based guidance for clinicians to devise personalized and effective treatment plans for patients with RCCT. It is recommended that patients with mild-to-moderate symptoms primarily receive nonsurgical treatments, notably comprehensive PT and RSWT+PT, as these methods significantly aid in functional recovery and pain reduction. For patients with severe symptoms who do not respond well to nonsurgical treatments, ABD+ASD or ABD alone are suggested as more definitive treatment options. In addition, UGN+SAI is an effective method for the removal of calcific deposits.

## Conclusion

This systematic review and NMA comprehensively evaluated both nonsurgical and surgical treatment approaches for RCCT. The results indicated that, in nonsurgical interventions, comprehensive PT approaches demonstrated the most effective results in functional improvement, followed by ESWT-H+PT. For pain relief, RSWT+PT showed the best performance, followed by ESWT-M, although no significant statistical differences were observed among the treatments. In surgical treatments, ABD alone demonstrated similar clinical effects to ABD + ASD in both functional improvement and pain relief. Regarding the resolution of calcific deposits, UGN+SAI showed better therapeutic potential. However, it is important to note that there is currently no direct data to compare the effectiveness of operative versus nonoperative treatments for RCCT. Considering the limitations in statistical significance and existing evidence, future research should involve more high-quality multicenter RCTs to validate the relative efficacy of these treatment methods.

## Supplementary materials



## ICMJE Statement of Interest

The authors declare that there is no conflict of interest that could be perceived as prejudicing the impartiality of the work reported.

## Funding Statement

This work was supported by the National Key R&D Program of China (2023YFC3603400); National Natural Science Foundation of China (82072506, 92268115); Hunan Provincial Science Fund for Distinguished Young Scholars (2024JJ2089); National Clinical Research Center for Geriatric Disorders (Xiangya Hospital, 2021KF02).

## Author contribution statement

In the writing of this manuscript, YMY was responsible for the conceptualization, data curation, visualization and writing of the original draft. GY also engaged in conceptualization and wrote the original draft in addition to performing formal analysis and developing the methodology. SDJ contributed to data curation and investigation. BZJ managed the project administration and validation. HFJ provided software support and participated in the review and editing of the writing. PYT was involved in methodology formulation and visualization. HZL oversaw the supervision and validation of the project. Finally, YSL acquired funding, supervised the project and contributed to the writing by reviewing and editing. BBL contributed to the reviewing process.
