# Supplementary material for: Treatments for rotator cuff calcific tendinitis: a systematic review and network meta-analysis of randomized-controlled trials
Source: EFORT Open Rev. 2025 Jun 30;10(7):520–33. doi: 10.1530/EOR-2024-0078 (PMC12232402; doi:10.1530/EOR-2024-0078)
Supplement: Supplementary file 1 [file supplementary_materials.pdf]

Appendix

Table S1. Retrieval Strategies..1

Table S2. Risk of bias of included randomized controlled trials.................. 4

Table S3. League table showing network meta-analysis estimates improvement in non-surgical treatment function 5

Table S4. League table showing network meta-analysis estimates of pain relief for non-surgical treatment... 6

Table S5. League table showing network meta-analysis estimates improvement in operative treatment function.................................... 7

Table S6. League table showing network meta-analysis estimates of pain relief for operative treatment 8

Table S7. SUCRA Rankings 9

Table S8. Summary of resolution of calcific deposit 10

Figure S1. Trace and density plots...11

Figure S2. Shrinkage plots 12

References...14

Table S1. Retrieval Strategies

Serial number	Search strategy in PubMed
#1	((((((("Rotator Cuff"[Mesh]) OR (Cuff, Rotator[Title/Abstract])) OR (Rotator Cuffs[Title/Abstract])) OR (Teres Minor[Title/Abstract])) OR (Subscapularis[Title/Abstract])) OR (Infraspinatus[Title/Abstract])) OR (Supraspinatus[Title/Abstract])) OR (shoulder[Title/Abstract])
#2	((((((((((((((("Calcinosis"[Mesh])) OR (Calcinosis[Title/Abstract])) OR (Calcific[Title/Abstract])) OR (Calcified[Title/Abstract])) OR (Calcification[Title/Abstract])) OR (Calcify[Title/Abstract])) OR (Calcinoses[Title/Abstract])) OR (Calcified deposit[Title/Abstract])) OR (Mineralization[Title/Abstract])) OR (Calcified tissue[Title/Abstract])) OR (Calcified lesion[Title/Abstract])) OR (Ossification[Title/Abstract])) OR (Microcalcinosis[Title/Abstract])) OR (Microcalcifications[Title/Abstract])) OR (Microcalcinoses[Title/Abstract])) OR (Microcalcification[Title/Abstract])) OR (Hypercalcification[Title/Abstract])
#3	((((((((((("Tendinopathy"[Mesh]) OR (Tendinopathy[Title/Abstract])) OR (Tendinitis[Title/Abstract])) OR (Tendinitides[Title/Abstract])) OR (Tendinosis[Title/Abstract])) OR (Tendinoses[Title/Abstract])) OR (Tendonosis[Title/Abstract])) OR (Tendonoses[Title/Abstract])) OR (Tendinopathies[Title/Abstract])) OR (Tendonopathy[Title/Abstract])) OR (Tendonopathies[Title/Abstract])) OR (Tendonitis[Title/Abstract])) OR (Tendonitides[Title/Abstract])
#4	#1 AND (#2 OR #3)
#5	((((((((((((random*[Title])) OR (factorial[Title/Abstract])) OR (crossover[Title/Abstract])) OR (placebo[Title/Abstract])) OR (control[Title/Abstract])) OR (trial[Title/Abstract])) OR (group[Title/Abstract])) OR (randomized controlled trial[Title/Abstract])) OR ("crossover procedure"[MeSH])) OR ("single blind procedure"[MeSH])) OR ("double blind procedure"[MeSH])) OR ("randomized controlled trial[Publication Type])
#6	#4 AND #5

Serial number	Search strategy in Web of Science
#1	((((((TS=(Rotator Cuff)) OR TS=((Rotator Cuffs)) OR TS=(Teres Minor)) OR TS=(Subscapularis)) OR TS=(Infraspinatus)) OR TS=(Supraspinatus)) OR TS=(shoulder)
#2	((((((((((((((TS=(Calcinosis)) OR TS=(Calcific)) OR TS=(Calcified)) OR TS=(Calcification)) OR TS=(Calcify)) OR TS=(Calcinoses)) OR TS=(Calcified deposit)) OR TS=(Mineralization)) OR TS=(Calcified tissue)) OR TS=(Calcified lesion)) OR TS=(Ossification)) OR TS=(Microcalcinosis)) OR TS=(Microcalcifications)) OR TS=(Microcalcinoses)) OR TS=(Microcalcification)) OR TS=(Hypercalcification)
#3	(((((((((((TS=(Tendinopathy)) OR TS=(Tendinitis)) OR TS=(Tendinitides)) OR TS=(Tendinosis)) OR TS=(Tendinoses)) OR TS=(Tendonosis)) OR TS=(Tendonoses)) OR TS=(Tendinopathies)) OR TS=(Tendonopathy)) OR TS=(Tendonopathies)) OR TS=(Tendonitis)) OR TS=(Tendonitides)
#4	#1 AND (#2 OR #3)
#5	(((((((((((TS=(random)) OR TS=(factorial)) OR TS=(crossover)) OR TS=(placebo)) OR TS=(control)) OR TS=(trial)) OR TS=(group)) OR TS=(randomized controlled trial)) OR

	TS=(crossover procedure)) OR TS=(single blind procedure)) OR TS=(double blind procedure)) OR TS=(randomized controlled trial)
#6	#4 AND #5

**Serial number**	**Search strategy in Embase**
#1	'rotator cuff'/exp
#2	'rotator cuff':ti,ab,kw OR 'rotator cuffs':ti,ab,kw OR 'teres minor':ti,ab,kw OR subscapularis:ti,ab,kw OR infraspinatus:ti,ab,kw OR supraspinatus:ti,ab,kw OR shoulder:ti,ab,kw
#3	#1 OR #2
#4	'calcinosis'/exp
#5	calcinosis:ti,ab,kw OR calcific:ti,ab,kw OR calcified:ti,ab,kw OR calcification:ti,ab,kw OR calcify:ti,ab,kw OR calcinoses:ti,ab,kw OR 'calcified deposit':ti,ab,kw OR mineralization:ti,ab,kw OR 'calcified tissue':ti,ab,kw OR 'calcified lesion':ti,ab,kw OR ossification:ti,ab,kw OR microcalcinosis:ti,ab,kw OR microcalcifications:ti,ab,kw OR microcalcinoses:ti,ab,kw OR microcalcification:ti,ab,kw OR hypercalcification:ti,ab,kw
#6	#4 OR #5
#7	'tendinitis'/exp
#8	tendinopathy:ti,ab,kw OR tendinitis:ti,ab,kw OR tendinosis:ti,ab,kw OR tendinoses:ti,ab,kw OR tendonosis:ti,ab,kw OR tendonoses:ti,ab,kw OR tendinitides:ti,ab,kw OR tendinopathies:ti,ab,kw OR tendonopathy:ti,ab,kw OR tendonopathies:ti,ab,kw OR tendonitis:ti,ab,kw OR tendonitides:ti,ab,kw
#9	#7 OR #8
#10	#3 AND (#6 OR #9)
#11	'crossover procedure'/exp OR 'single blind procedure'/exp OR 'double blind procedure'/exp
#12	random:ti,ab,kw OR factorial:ti,ab,kw OR crossover:ti,ab,kw OR placebo:ti,ab,kw OR control:ti,ab,kw OR trial:ti,ab,kw OR group:ti,ab,kw OR 'randomized controlled trial':ti,ab,kw
#13	#10 OR #11
#14	#10 AND #13

**Serial number**	**Search strategy in Cochrane Library**
#1	MeSH descriptor: [Rotator Cuff] explode all trees
#2	(Rotator Cuff):ti,ab,kw OR (Rotator Cuffs):ti,ab,kw OR (Teres Minor):ti,ab,kw OR (Subscapularis):ti,ab,kw OR (Infraspinatus):ti,ab,kw
#3	(Supraspinatus):ti,ab,kw OR (shoulder):ti,ab,kw
#4	#1 or #2 or #3
#5	MeSH descriptor: [Calcinosis] explode all trees
#6	(Calcinosis):ti,ab,kw OR (Calcific):ti,ab,kw OR (Calcified):ti,ab,kw OR (Calcification):ti,ab,kw OR (Calcify):ti,ab,kw
#7	(Calcinoses):ti,ab,kw OR (Calcified deposit):ti,ab,kw OR (Mineralization):ti,ab,kw OR (Calcified tissue):ti,ab,kw OR (Calcified lesion):ti,ab,kw
#8	(Ossification):ti,ab,kw OR (Microcalcinosis):ti,ab,kw OR (Microcalcifications):ti,ab,kw OR

	(Microcalcinoses):ti,ab,kw OR (Microcalcification):ti,ab,kw
#9	(Hypercalcification):ti,ab,kw
#10	#5 or #6 or #7 or #8 or #9
#11	MeSH descriptor: [Tendinopathy] explode all trees
#12	(Tendinopathy):ti,ab,kw OR (Tendinitis):ti,ab,kw OR (Tendinitides):ti,ab,kw OR (Tendinosis):ti,ab,kw OR (Tendinoses):ti,ab,kw
#13	(Tendonosis):ti,ab,kw OR (Tendonoses):ti,ab,kw OR (Tendinopathies):ti,ab,kw OR (Tendonopathy):ti,ab,kw OR (Tendonopathies):ti,ab,kw
#14	(Tendonitis):ti,ab,kw OR (Tendonitides):ti,ab,kw
#15	#11 or #12 or #13 or #14
#16	#4 and (#10 or #15)
#17	MeSH descriptor: [Randomized Controlled Trial] explode all trees
#18	(random):ti,ab,kw OR (factorial):ti,ab,kw OR (crossover):ti,ab,kw OR (placebo):ti,ab,kw OR (control):ti,ab,kw
#19	(trial):ti,ab,kw OR (group):ti,ab,kw OR (randomized controlled trial):ti,ab,kw OR (crossover procedure):ti,ab,kw OR (single blind procedure):ti,ab,kw
#20	(double blind procedure):ti,ab,kw
#21	#17 or #18 or #19 # 20
#22	#16 and #21

Table S2. Risk of bias of included randomized controlled trials

Study	Randomization process	Deviations from intended interventions	Missing outcome data	Measurement of the outcome	Selection of the reported result	Overall Bias
Moosmayer(2023)[1]	Low	Low	Low	Low	Low	Low
Verstraelen(2022)[2]	Some concerns	Low	Low	Low	Low	Some concerns
Fatima(2022)[3]	Low	Low	Low	Low	Low	Low
Kim(2022)[4]	Low	Low	Low	Low	Low	Low
Kuo(2022)[5]	Low	Low	Low	Low	Low	Low
Al-Khair(2021)[6]	Low	Low	Low	Low	Low	Low
Louwerens(2020)[7]	Low	Low	Low	Low	Low	Low
Duymaz(2019)[8]	Low	Low	Low	Low	Some concerns	Some concerns
Darrieutort-Laffite (2019)[9]	Low	Low	Low	Low	Low	Low
Papadopoulos(2019)[10]	Low	Low	Low	Low	Low	Low
Pieber(2018)[11]	Some concerns	High	Low	High	Low	High
De Boer(2017)[12]	Low	Low	Low	Low	Low	Low
de Witte(2017)[13]	Some concerns	Low	Low	Low	Low	Some concerns
Battaglia(2017)[14]	Some concerns	Low	Low	Some concerns	Low	Some concerns
Clement(2015)[15]	Low	Low	Low	Low	Low	Low
Kim(2014)[16]	Low	Low	Low	Low	Low	Low
Sabeti(2014)[17]	Low	Low	Low	Low	Low	Low
Kolk(2013)[18]	Low	Low	Low	Low	Low	Low
de Witte(2013)[19]	Low	Low	Low	Low	Low	Low
Ioppolo(2012)[20]	Low	Low	Low	Low	Low	Low
Tornese(2011)[21]	Low	Low	Low	Low	Low	Low
Zhu(2008)[22]	Some concerns	Low	Low	Low	Low	Some concerns
Hsu(2008)[23]	Low	Low	Low	Low	Low	Low
Albert(2007)[24]	Low	Low	Low	Low	Low	Low
Sabeti(2006)[25]	Low	Low	Low	Low	Low	Low
Cacchio(2006)[26]	Low	Low	Low	Low	Low	Low
Sabeti-Aschraf(2005)[27]	Low	Low	Low	Low	Low	Low
Krasny(2005)[28]	Low	Low	Low	Low	Low	Low
Pleiner(2004)[29]	Some concerns	Low	Low	High	Low	High
Gerdesmeyer(2003)[30]	Low	Low	Low	Low	Low	Low
Pan(2003)[31]	Some concerns	Low	Low	Some concerns	Low	Some concerns
Cosentino(2003)[32]	Low	Low	Low	Low	Low	Low
Perlick(2003)[33]	Some concerns	Some concerns	Low	High	Low	High

Table S3. League table showing network meta-analysis estimates improvement in non-surgical treatment function

**ESWT+H**			**N = 2, -0.53 (-6.51, 5.38)**				**11.05 (0.46, 21.52)**			**N = 1, -1.22 (-9.65, 7.1)**		**N = 1, 0.41 (-7.94, 8.79)**	**N= 1, 0.32 (-8.12, 8.69)**	**-**
-11.14 (-24.59, 2.4)	**ESWT-H+PT**					**-16.98 (-31.81, -2.35)**	**N = 1, -0.06 (-8.45, 8.22)**		**-11.24 (-21.77, -0.79)**				**-10.81 (-21.26, -0.31)**	**-17.11 (-34.23, -0.24)**
5.53 (-10.11, 21.44)	16.65 (-0.26, 33.76)	**ESWT-HIR**				**N = 1, -0.32 (-8.74, 8.11)**	**16.58 (1.9, 31.43)**							
0.53 (-5.38, 6.51)	11.66 (-3.13, 26.38)	-5 (-22, 11.73)	**ESWT-L**	**N = 2, 0.98 (-4.93, 6.96)**										
-0.46 (-8.81, 7.94)	10.7 (-5.28, 26.52)	-5.97 (-24.06, 11.76)	-0.98 (-6.96, 4.93)	**ESWT-M**										
-0.1 (-14.63, 14.49)	11.03 (-4.8, 26.89)	-5.62 (-19.03, 7.71)	-0.63 (-16.32, 15.17)	0.35 (-16.38, 17.19)	**FSWT**			**N = 2, -0.67 (-9.02, 7.75)**						
5.85 (-7.34, 19.22)	16.98 (2.35, 31.81)	0.32 (-8.11, 8.74)	5.32 (-9.11, 20.01)	6.31 (-9.31, 22.14)	5.95 (-4.31, 16.3)	**NON**	**16.91 (4.83, 29.08)**	**N = 2, 5.28 (-0.61, 11.35)**						**N = 1, -0.14 (-8.49, 8.24)**
-11.05 (-21.52, -0.46)	0.06 (-8.22, 8.45)	-16.58 (-31.43, -1.9)	-11.59 (-23.69, 0.54)	-10.61 (-24.13, 2.88)	-10.96 (-24.48, 2.54)	-16.91 (-29.08, -4.83)	**PT**	**-11.62 (-22.14, -1.04)**	**N = 1, -11.18 (-17.51, -4.83)**				**N = 1, -10.74 (-17.11, -4.36)**	**-17.05 (-31.84, -2.4)**
0.58 (-11.26, 12.41)	11.71 (-1.71, 25.16)	-4.94 (-15.38, 5.32)	0.04 (-13.19, 13.35)	1.01 (-13.57, 15.57)	0.67 (-7.75, 9.02)	-5.28 (-11.35, 0.61)	11.62 (1.04, 22.14)	**RSWT**						
0.12 (-9.35, 9.76)	11.24 (0.79, 21.77)	-5.39 (-19.55, 8.54)	-0.41 (-11.58, 10.9)	0.58 (-12.11, 13.32)	0.22 (-12.5, 12.94)	-5.73 (-17.08, 5.43)	11.18 (4.83, 17.51)	-0.44 (-9.98, 9.1)	**SAI**				**N = 3, 0.45 (-4.04, 4.96)**	
1.22 (-7.1, 9.65)	12.36 (-3.56, 28.24)	-4.29 (-22.35, 13.5)	0.69 (-9.55, 10.93)	1.67 (-10.16, 13.53)	1.33 (-15.53, 18.11)	-4.62 (-20.46, 11.05)	12.27 (-1.2, 25.73)	0.68 (-13.94, 15.17)	1.1 (-11.62, 13.75)	**TENS**				
0.29 (-14.23, 14.84)	11.42 (-4.47, 27.23)	-5.24 (-18.69, 8.01)	-0.23 (-15.92, 15.44)	0.75 (-16.06, 17.48)	0.4 (-11.45, 12.17)	-5.56 (-15.99, 4.68)	11.36 (-2.11, 24.81)	-0.27 (-8.65, 8.15)	0.16 (-12.52, 12.82)	-0.93 (-17.8, 15.84)	**UGN**			
-0.41 (-8.79, 7.94)	10.73 (-5.2, 26.55)	-5.91 (-23.97, 11.82)	-0.94 (-11.14, 9.28)	0.03 (-11.73, 11.9)	-0.33 (-17.21, 16.34)	-6.26 (-22.13, 9.37)	10.66 (-2.8, 24)	-0.97 (-15.59, 13.47)	-0.5 (-13.2, 12.03)	-1.63 (-13.45, 10.15)	-0.71 (-17.53, 15.97)	**UGN+ESWT-H**		
-0.32 (-8.69, 8.12)	10.81 (0.31, 21.26)	-5.83 (-19.25, 7.38)	-0.85 (-11.14, 9.47)	0.13 (-11.7, 12.02)	-0.23 (-12.15, 11.67)	-6.18 (-16.59, 4.04)	10.74 (4.36, 17.11)	-0.9 (-9.29, 7.54)	-0.45 (-4.96, 4.04)	-1.54 (-13.43, 10.28)	-0.62 (-12.47, 11.3)	0.06 (-11.69, 11.98)	**UGN+SAI**	
5.99 (-9.69, 21.81)	17.11 (0.24, 34.23)	0.47 (-11.47, 12.37)	5.45 (-11.28, 22.35)	6.42 (-11.32, 24.33)	6.08 (-7.18, 19.44)	0.14 (-8.24, 8.49)	17.05 (2.4, 31.84)	5.42 (-4.81, 15.87)	5.85 (-8.12, 19.99)	4.77 (-13.03, 22.74)	5.7 (-7.52, 19.01)	6.41 (-11.39, 24.35)	6.31 (-6.89, 19.68)	**US**

Note: 1. Lower-left triangle presents the findings (SMD with 95% CI) of the network meta-analysis conducted using R 4.3.1. Upper-right triangle presents the findings (SMD with 95% CI) of the pair-wise meta-analyses conducted using STATA 17 and N refers to the numbers of RCTs which compared the 2 interventions directly. 3. A positive SMD favors the lower-right intervention; a negative SMD favors the upper-left intervention. 4.Statistically significant findings are shaded. ESWT, extracorporeal shock wave therapy; ESET - H, high - energy extracorporeal shock wave therapy; ESWT - HIR, ESWT - hyperextended internal rotation technique; ESWT - L, low - energy extracorporeal shock wave therapy; ESWT - M, middle - energy extracorporeal shock wave therapy; FSWT, focused shock wave therapy; NON, sham/placebo; PT, routine physical therapy; RSWT, radial shock wave therapy; SAI, subacromial corticosteroid injection; TENS, transcutaneous electric nerve stimulation; UGN, ultrasound - guided needling ; US, ultrasound therapy.

Table S4. League table showing network meta-analysis estimates of pain relief for non-surgical treatment

**ESWT-H**		N = 3, 0.55<br>(-1.69, 2.81)								N = 1, 0.96<br>(-2.95, 4.88)			N = 1, -0.44<br>(-4.3, 3.46)	
0.88<br>(-5.53, 7.24)	**ESWT-H+PT**					N = 1, 0.80<br>(-3.14, 4.72)								
-0.55<br>(-2.81, 1.69)	-1.44<br>(-8.15, 5.33)	**ESWT-L**	N = 2, -1.33<br>(-4.13, 1.44)											
0.78<br>(-2.79, 4.37)	-0.11<br>(-7.36, 7.25)	1.33<br>(-1.44, 4.13)	**ESWT-M**											
0.08<br>(-6.13, 6.27)	-0.81<br>(-7.76, 6.14)	0.64<br>(-5.96, 7.19)	-0.71<br>(-7.83, 6.42)	**FSWT**			N = 2, 0.3<br>(-2.46, 3.08)							
-2.12<br>(-8.34, 4.06)	-3<br>(-9.95, 3.93)	-1.56<br>(-8.14, 4.99)	-2.90<br>(-10.06, 4.2)	-2.20<br>(-6.12, 1.7)	**NON**		N = 2, -1.90<br>(-4.67, 0.86)							
0.08<br>(-4.93, 5.07)	-0.80<br>(-4.72, 3.14)	0.64<br>(-4.84, 6.12)	-0.70<br>(-6.85, 5.43)	0<br>(-5.74, 5.76)	2.20<br>(-3.52, 7.99)	**PT**		N = 1, -1.48<br>(-5.36, 2.42)	N = 1, -0.13<br>(-3.31, 3.04)				N = 1, -0.36<br>(-3.52, 2.84)	
-0.23<br>(-5.79, 5.31)	-1.11<br>(-7.46, 5.28)	0.32<br>(-5.64, 6.29)	-1.01<br>(-7.61, 5.55)	-0.30<br>(-3.08, 2.46)	1.90<br>(-0.86, 4.67)	-0.31<br>(-5.35, 4.72)	**RSWT**				N = 1, -0.63<br>(-4.55, 3.28)		N = 1, -0.66<br>(-4.61, 3.28)	
1.56<br>(-4.79, 7.89)	0.69<br>(-4.83, 6.21)	2.11<br>(-4.6, 8.85)	0.78<br>(-6.52, 8.04)	1.49<br>(-5.46, 8.44)	3.68<br>(-3.24,<br>10.67)	1.48<br>(-2.42, 5.36)	1.80<br>(-4.58, 8.19)	**RSWT+PT**						
0.22<br>(-4.82, 5.23)	-0.67<br>(-5.71, 4.4)	0.77<br>(-4.72, 6.26)	-0.56<br>(-6.72, 5.57)	0.14<br>(-5.62, 5.89)	2.33<br>(-3.42, 8.11)	0.13<br>(-3.04, 3.31)	0.44<br>(-4.6, 5.49)	-1.35<br>(-6.36, 3.68)	**SAI**				N = 1, -0.22<br>(-3.37, 2.97)	
-0.96<br>(-4.88, 2.95)	-1.83<br>(-9.34, 5.65)	-0.39<br>(-4.91, 4.09)	-1.73<br>(-7.07, 3.53)	-1.03<br>(-8.35, 6.29)	1.16<br>(-6.11, 8.5)	-1.03<br>(-7.42, 5.31)	-0.72<br>(-7.51, 6.08)	-2.51<br>(-9.98, 4.93)	-1.17<br>(-7.53, 5.17)	**TENS**				
0.39<br>(-6.39, 7.19)	-0.49<br>(-7.96, 7)	0.94<br>(-6.12, 8.09)	-0.39<br>(-8.01, 7.26)	0.32<br>(-4.45, 5.13)	2.52<br>(-2.26, 7.34)	0.32<br>(-6.07, 6.7)	0.63<br>(-3.28, 4.55)	-1.16<br>(-8.63, 6.29)	0.18<br>(-6.19, 6.59)	1.35<br>(-6.49, 9.19)	**UGN**	N = 1, -0.25<br>(-4.14, 3.65)		
0.64<br>(-7.16, 8.47)	-0.23<br>(-8.66, 8.22)	1.2<br>(-6.91, 9.33)	-0.13<br>(-8.72, 8.44)	0.57<br>(-5.56, 6.74)	2.77<br>(-3.37, 8.93)	0.57<br>(-6.88, 8.05)	0.87 (-4.61,<br>6.39)	-0.91<br>(-9.37, 7.55)	0.43<br>(-7.03, 7.92)	1.60<br>(-7.16,<br>10.29)	0.25<br>(-3.65, 4.14)	**UGN-A**		
0.44<br>(-3.46, 4.3)	-0.44<br>(-5.5, 4.59)	0.99<br>(-3.49, 5.47)	-0.34<br>(-5.64, 4.91)	0.36<br>(-4.46, 5.17)	2.55<br>(-2.24, 7.38)	0.36<br>(-2.84, 3.52)	0.66<br>(-3.28, 4.61)	-1.13<br>(-6.16, 3.89)	0.22<br>(-2.97, 3.37)	1.39<br>(-4.14, 6.88)	0.04<br>(-5.51, 5.56)	-0.21<br>(-7, 6.51)	**UGN+SAI**	N = 1, 0<br>(-3.86, 3.86)
0.42<br>(-5.06, 5.9)	-0.44<br>(-6.81, 5.88)	0.98<br>(-4.93, 6.89)	-0.35<br>(-6.93, 6.16)	0.35<br>(-5.83, 6.52)	2.54<br>(-3.65, 8.75)	0.35<br>(-4.66, 5.34)	0.66<br>(-4.87, 6.18)	-1.13<br>(-7.46, 5.17)	0.22<br>(-4.8, 5.23)	1.38<br>(-5.34, 8.09)	0.03<br>(-6.79, 6.81)	-0.22<br>(-8.03, 7.6)	0<br>(-3.86, 3.86)	**UGN+SSI**

Note: 1. Lower-left triangle presents the findings (SMD with 95% CI) of the network meta-analysis conducted using R 4.3.1. Upper-right triangle presents the findings (SMD with 95% CI) of the pair-wise meta-analyses conducted using STATA 17 and N refers to the numbers of RCTs which compared the 2 interventions directly. 3. A positive SMD favors the lower-right intervention; a negative SMD favors the upper-left intervention. ESWT, extracorporeal shock wave therapy; ESET - H, high - energy extracorporeal shock wave therapy; ESWT - L, low - energy extracorporeal shock wave therapy; ESWT - M, middle - energy extracorporeal shock wave therapy; FSWT, focused shock wave therapy; NON, sham/placebo; PT, routine physical therapy; RSWT, radial shock wave therapy; SAI, subacromial corticosteroid injection; SSI, subacromial saline injection; TENS, transcutaneous electric nerve stimulation; UGN, ultrasound - guided needling ; UGN - A, UGN - with aspiration.

Table S5. League table showing network meta-analysis estimates improvement in operative treatment function

**ABD**	N = 2, 0.01 (-0.57, 0.54)	N = 1, -0.05 (-0.78, 0.64)	N = 1, -0.61 (-1.73, 0.51)
-0.01 (-0.54, 0.57)	**ABD+ASD**	N = 1, -0.06 (-0.76, 0.65)	
0.05 (-0.64, 0.78)	0.06 (-0.65, 0.76)	**ASD**	
0.61 (-0.51, 1.73)	0.62 (-0.65, 1.86)	0.55 (-0.79, 1.88)	**us-ABD**

Note: 1. Lower-left triangle presents the findings (SMD with 95% CI) of the network meta-analysis conducted using R 4.3.1. Upper-right triangle presents the findings (SMD with 95% CI) of the pair-wise meta-analyses conducted using STATA 17 and N refers to the numbers of RCTs which compared the 2 interventions directly. 3. A positive SMD favors the lower-right intervention; a negative SMD favors the upper-left intervention. ABD, arthroscopic bursectomy debridement of rotator cuff; ASD, arthroscopic subacromial decompression; us - ABD, ABD of ultrasound positioning.

Table S6. League table showing network meta-analysis estimates of pain relief for operative treatment

**ABD**	N = 2, 0.02 (-0.43, 0.51)	N = 1, 0.42 (-0.18, 1.04)	N = 1, 0.39 (-0.61, 1.41)
-0.02 (-0.51, 0.43)	**ABD+ASD**	N = 1, 0.4 (-0.21, 1)	
-0.42 (-1.04, 0.18)	-0.4 (-1, 0.21)	**ASD**	
-0.39 (-1.41, 0.61)	-0.37 (-1.48, 0.76)	0.03 (-1.14, 1.2)	**us-ABD**

Note: 1. Lower-left triangle presents the findings (SMD with 95% CI) of the network meta-analysis conducted using R 4.3.1. Upper-right triangle presents the findings (SMD with 95% CI) of the pair-wise meta-analyses conducted using STATA 17 and N refers to the numbers of RCTs which compared the 2 interventions directly. 3. A positive SMD favors the lower-right intervention; a negative SMD favors the upper-left intervention. ABD, arthroscopic bursectomy debridement of rotator cuff; ASD, arthroscopic subacromial decompression; us - ABD, ABD of ultrasound positioning.

Table S7. SUCRA Rankings

NFunctionScore			NPainScore		
**Treatment**	**Possibility**	**Rank**	**Treatment**	**Possibility**	**Rank**
PT	93.98%	1	RSWT+PT	70.72%	1
ESWT-H+PT	92.42%	2	ESWT-M	61.51%	2
UGN+SAI	54.48%	3	ESWT-H+PT	61.08%	3
ESWT-M	54.05%	4	UGN-A	57.45%	4
UGN+ESWT-H	53.31%	5	UGN+SAI	56.61%	5
FSWT	52.81%	6	UGN+SSI	54.98%	6
ESWT-H	51.30%	7	UGN	54.76%	7
UGN	50.35%	8	SAI	51.18%	8
SAI	50.08%	9	FSWT	50.28%	9
RSWT	49.13%	10	ESWT-H	48.90%	10
ESWT-L	46.35%	11	PT	48.00%	11
TENS	42.52%	12	RSWT	44.43%	12
ESWT-HIR	22.13%	13	ESWT-L	37.83%	13
US	19.89%	14	TENS	34.50%	14
NON	17.20%	15	NON	17.77%	15
**SFunctionScore**			**SPainScore**		
**Treatment**	**Possibility**	**Rank**	**Treatment**	**Possibility**	**Rank**
ABD+ASD	64.49%	1	ABD	74.84%	1
ABD	63.88%	2	ABD+ASD	70.30%	2
ASD	55.38%	3	us-ABD	33.34%	3
us-ABD	16.25%	4	ASD	21.52%	4

ABD, arthroscopic bursectomy debridement of rotator cuff; ASD, arthroscopic subacromial decompression; ESWT, extracorporeal shock wave therapy; ESET - H, high - energy extracorporeal shock wave therapy; ESWT - HIR, ESWT - hyperextended internal rotation technique; ESWT - L, low - energy extracorporeal shock wave therapy; ESWT - M, middle - energy extracorporeal shock wave therapy; FSWT, focused shock wave therapy; NFunctionScore, non-surgical treatment function score; NON, sham/placebo; NPainScore, non-surgical treatment pain score; PT, routine physical therapy; RSWT, radial shock wave therapy; SAI, subacromial corticosteroid injection; SFunctionScore, surgical treatment function score; SPainScore, surgical treatment pain score; SSI, subacromial saline injection; SUCRA, Surface Under the Cumulative Ranking; TENS, transcutaneous electric nerve stimulation; UGN, ultrasound - guided needling ; UGN - A, UGN - with aspiration; US, ultrasound therapy; us - ABD, ABD of ultrasound positioning.

Table S8. Summary of resolution of calcific deposit

Study	follow-up (mo)	Intervention A	Sample size, n	Deposit resolution	Mean size decrease, mm	Intervention B	Sample size, n	Deposit resolution	Mean size decrease, mm
Moosmayer (2023) [1]	4	UGN+SAI	72	Complete:33		SAI	70	Complete:6	
Moosmayer (2023) [1]	4	UGN+SAI	72	Complete:33		PT	66	Complete:0	
Kuo(2022) [5]	3	UGN	21	Complete:2		RSWT	20	Complete:2	
				Partial:19				Partial:16	
Al-Khair(2021) [6]	3	FSWT	15	Complete:3		RSWT	15	Complete:4	
				Partial:12				Partial:11	
Louwerens(2020) [7]	6	ESWT-H	43	Complete:4		UGN+SAI	43	Complete:27	
				Partial:10				Partial:13	
				Unchanged:17				Unchanged:0	
Darrieutort-Laffite (2019) [9]	12	UGN+SAI	62	Complete:46		UGN+SSI	59	Complete:49	
				Partial:7				Partial:8	
				Unchanged:3				Unchanged:2	
Papadopoulos (2019) [10]	0.75	EAU+NSAIDs	20	Complete:15	-13	NON	20	Complete:8	-9.20
De Boer(2017) [12]	1.5	UGN+SAI	11	Complete:5		RSWT	14	Complete:12	
Battaglia(2017) [14]	6	UGN+TA	20	Complete:20		UGN+MA	20	Complete:12	
Kim(2014) [16]	12	UGN+SAI	25	Complete:18	-14.35	ESWT-H	29	Complete:12	-5.4
				Partial:3				Partial:5	
				Unchanged:4				Unchanged:12	
Sabeti(2014) [17]	9	ABD	10	Complete:9		us-ABD	10	Complete:8	
				Partial:1				Partial:0	
de Witte(2013) [19]	12	UGN+SAI	23	Complete:13	-11.6±6.4	SAI	25	Complete:6	-5.1±5.7
				Partial:22				Partial:17	
Ioppolo(2012) [20]	6	ESWT-M	23	Complete:11		ESWT-L	23	Complete:12	
Tornese(2011) [21]	3	NON	17	Complete:16		ESWT-HIR	18	Complete:12	
				Unchanged:11				Unchanged:6	
Hsu(2008) [23]	12	ESWT-H	33	Complete:7		NON	13	Partial:2	
				Partial:11				Unchanged:11	
				Unchanged:15					
Albert(2007) [24]	3	ESWT-H	40	Complete:6		ESWT-L	40	Complete:2	
				Partial:3				Partial:5	
Sabeti(2006) [25]	3	ESWT-L	21	Complete:4		ESWT-M	23	Complete:5	
				Partial:3				Partial:9	
Cacchio(2006) [26]	6	RSWT	45		-20.45	NON	45		-0.85
Sabeti-Aschraf (2005) [27]	3	ESWT-feedback	25	Complete:1		ESWT-navigation	25	Complete:6	
				Partial:12				Partial:9	
				Unchanged:12				Unchanged:20	
Krasny(2005) [28]	3	UGN+ ESWT-H	40	Complete:24		ESWT-H	40	Complete:13	
				Partial:10				Partial:14	
				Unchanged:6				Unchanged:13	
Pleiner(2004) [29]	3	ESWT-H	20	Complete:4		ESWT-L	18	Complete:2	
				Partial:8				Partial:3	
Gerdesmeyer (2003) [30]	12	ESWT-H	48		-162 mm^2	ESWT-L	48		-91.5 mm^2
Cosentino(2003) [32]	6	ESWT-H	35	Complete:11		NON	35	Complete:0	
				Partial:14				Partial:0	
Perlick(2003) [33]	12	ESWT-H	40	Complete:14		ESWT-L	40	Complete:6	
				Partial:8				Partial:9	
				Unchanged:18				Unchanged:25	

ABD, arthroscopic bursectomy debridement of rotator cuff; EAU, electroacupuncture; ESWT, extracorporeal shock wave therapy; ESET - H, high - energy extracorporeal shock wave therapy; ESWT - HIR, ESWT - hyperextended internal rotation technique; ESWT - L, low - energy extracorporeal shock wave therapy; ESWT - M, middle - energy extracorporeal shock wave therapy; FSWT, focused shock wave therapy; MA, methylprednisolone acetate; NON, sham/placebo; NSAIDs, nonsteroidal anti - inflammatory drugs; PT, routine physical therapy; RSWT, radial shock wave therapy; SAI, subacromial corticosteroid injection; SSI, subacromial saline injection; TA, triamcinolone acetonide; UGN, ultrasound - guided needling ; us - ABD, ABD of ultrasound positioning.

Figure S1. Trace and density plots

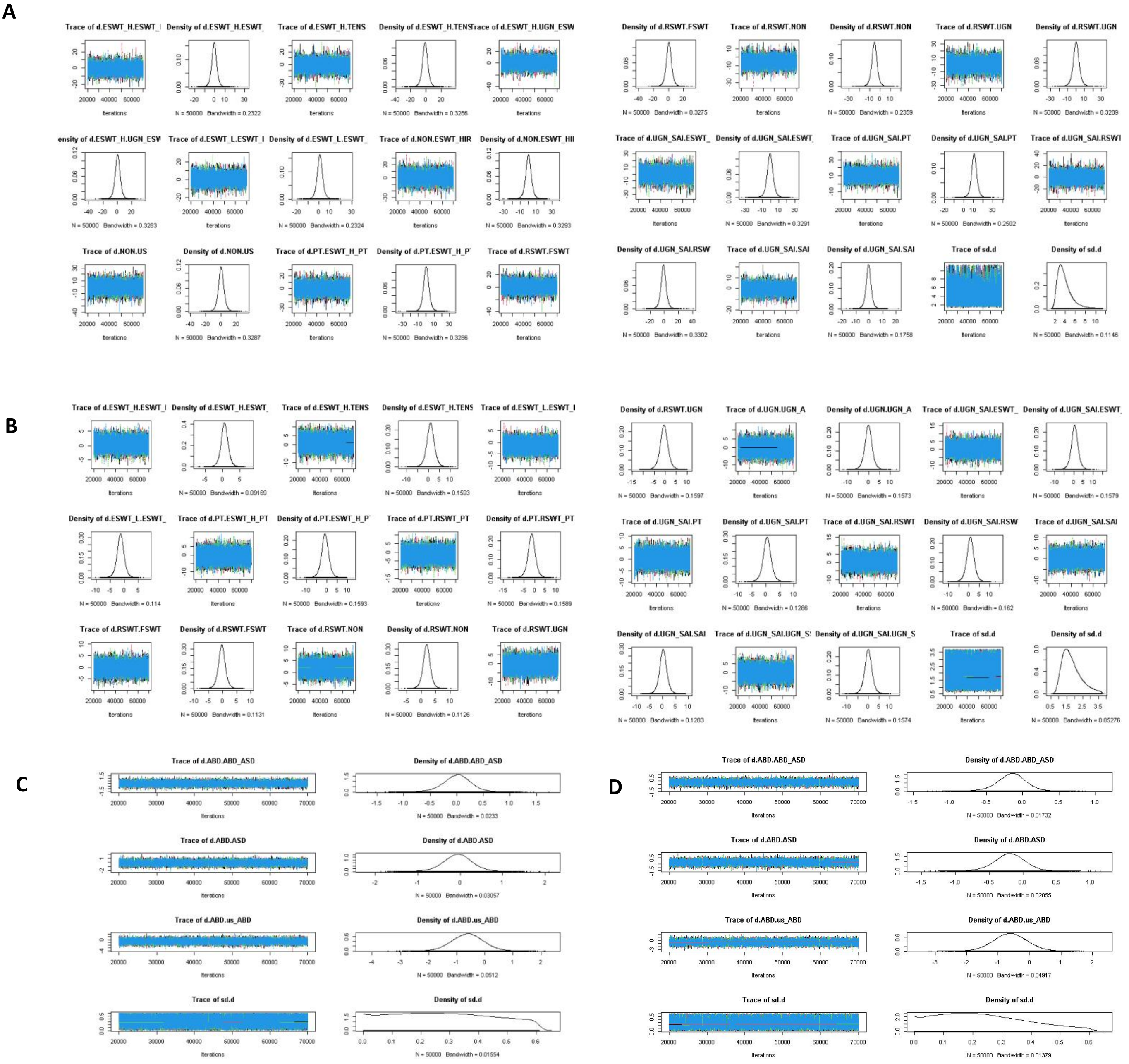

(A)Trace and density plot of non-surgical treatments (function score). (B) Trace and density plot of non-surgical treatments (pain score). (C) Trace and density plot of surgical treatments (function score). (D) Trace and density plot of surgical treatments (pain score). ABD, arthroscopic bursectomy debridement of rotator cuff; ASD, arthroscopic subacromial decompression; ESWT, extracorporeal shock wave therapy; ESET - H, high - energy extracorporeal shock wave therapy; ESWT - HIR, ESWT - hyperextended internal rotation technique; ESWT - L, low - energy extracorporeal shock wave therapy; ESWT - M, middle - energy extracorporeal shock wave therapy; FSWT, focused shock wave therapy; NON, sham/placebo; PT, routine physical therapy; RSWT, radial shock wave therapy; SAI, subacromial corticosteroid injection; SSI, subacromial saline injection; TENS, transcutaneous electric nerve stimulation; UGN, ultrasound - guided needling; UGN - A, UGN - with aspiration; US, ultrasound therapy; us - ABD, ABD of ultrasound positioning.

Figure S2. Shrinkage plots

A

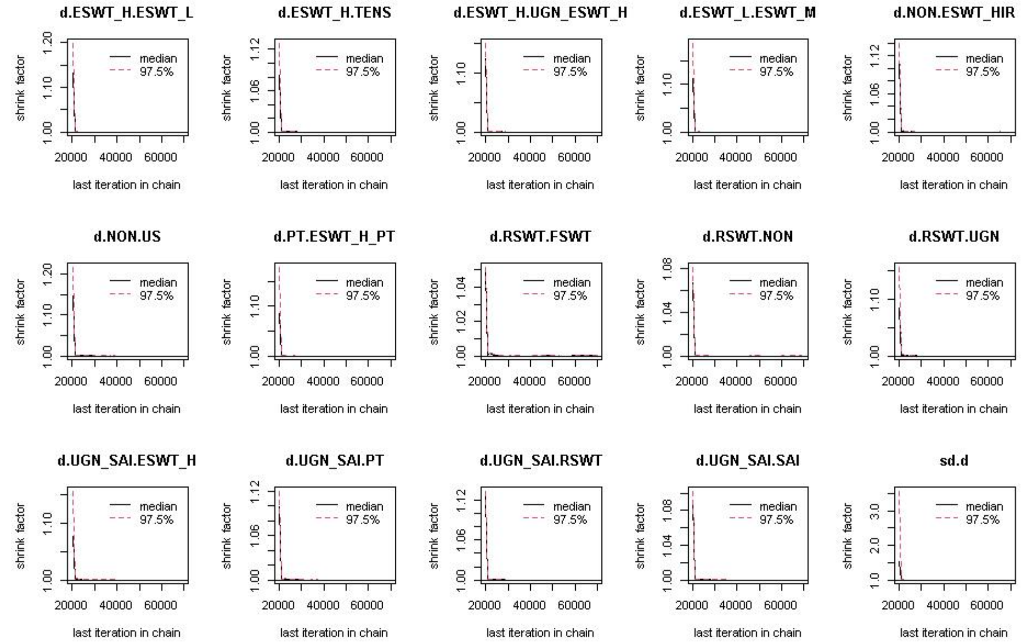

B

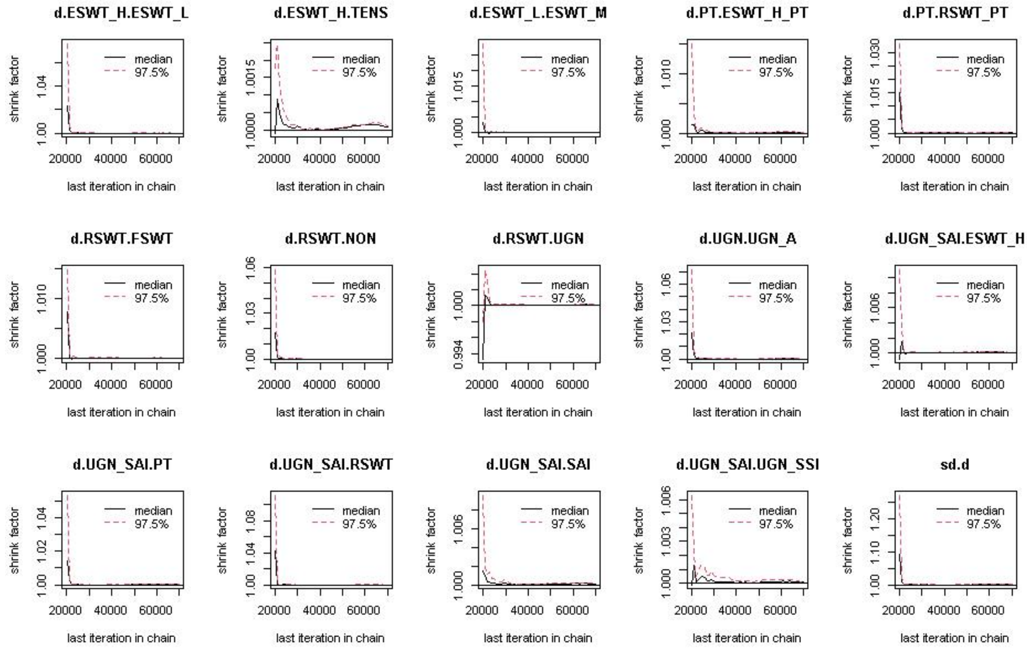

C

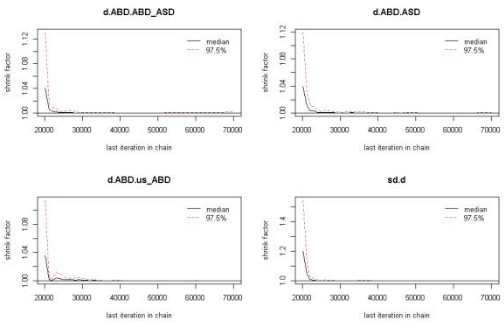

D

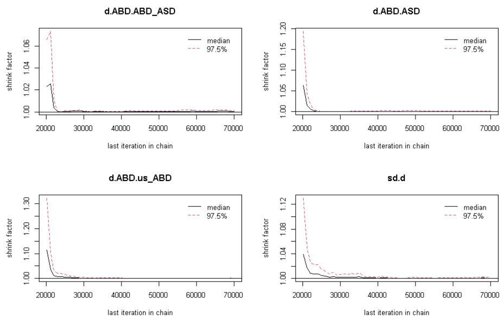

(A) Shrinkage plot of non-surgical treatments (function score). (B) Shrinkage plot of non-surgical treatments (pain score). (C) Shrinkage plot of surgical treatments (function score). (D) Shrinkage plot of surgical treatments (pain score). ABD, arthroscopic bursectomy debridement of rotator cuff; ASD, arthroscopic subacromial decompression; ESWT, extracorporeal shock wave therapy; ESET - H, high - energy extracorporeal shock wave therapy; ESWT - HIR, ESWT - hyperextended internal rotation technique; ESWT - L, low - energy extracorporeal shock wave therapy; ESWT - M, middle - energy extracorporeal shock wave therapy; FSWT, focused shock wave therapy; NON, sham/placebo; PT, routine physical therapy; RSWT, radial shock wave therapy; SAI, subacromial corticosteroid injection; SSI, subacromial saline injection; TENS, transcutaneous electric nerve stimulation; UGN, ultrasound - guided needling; UGN - A, UGN - with aspiration; US, ultrasound therapy; us - ABD, ABD of ultrasound positioning.

References

1. Moosmayer S, Ekeberg OM, Hallgren HB, et al. Ultrasound guided lavage with corticosteroid injection versus sham lavage with and without corticosteroid injection for calcific tendinopathy of shoulder: randomised double blinded multi-arm study. *BMJ.* 2023.
2. Verstraelen F, Schotanus M, Klemann-Harings S, Lambers Heerspink O, Jansen E. Comparison of clinical and radiological outcomes after three different surgical treatments for resistant calcifying tendinitis of the shoulder: a short-term randomized controlled trial. *J Orthop Surg Res.* 2022;17(1):480.
3. Fatima A, Ahmad A, Gilani SA, Darain H, Kazmi S, Hanif K. Effects of High-Energy Extracorporeal Shockwave Therapy on Pain, Functional Disability, Quality of Life, and Ultrasonographic Changes in Patients with Calcified Rotator Cuff Tendinopathy. *Biomed Res Int.* 2022;2022:1230857.
4. Kim J, Oh C, Yoo J, Yim J. Applying Focused and Radial Shock Wave for Calcific Tendinitis of the Shoulder : Randomized Controlled Study. *Physical Therapy Rehabilitation Science.* 2022;11(3):356-362.
5. Kuo YC, Hsu WC, Lin YJ, Lin YT, Chen YR, Hsieh LF. Comparison of the effects of ultrasound- guided needle puncture, radial shock wave therapy, and combined treatments on calcific tendinitis of the shoulder: A single-blind randomized controlled trial. *J Back Musculoskelet Rehabil.* 2022;35(5):1065-1074.
6. Abo Al-Khair MA, El Khouly RM, Khodair SA, Al Sattar Elsergany MA, Hussein MI, Eldin Mowafy ME. Focused, radial and combined shock wave therapy in treatment of calcific shoulder tendinopathy. *Phys Sportsmed.* 2021;49(4):480-487.
7. Louwerens JKG, Sierevelt IN, Kramer ET, et al. Comparing Ultrasound-Guided Needling Combined With a Subacromial Corticosteroid Injection Versus High-Energy Extracorporeal Shockwave Therapy for Calcific Tendinitis of the Rotator Cuff: A Randomized Controlled Trial. *Arthroscopy.* 2020;36(7):1823-1833 e1821.
8. Duymaz T, Sindel D. Comparison of Radial Extracorporeal Shock Wave Therapy and Traditional Physiotherapy in Rotator Cuff Calcific Tendinitis Treatment. *Arch Rheumatol.* 2019;34(3):281-287.
9. Darrieutort-Laffite C, Varin S, Coiffier G, et al. Are corticosteroid injections needed after needling and lavage of calcific tendinitis? Randomised, double-blind, non-inferiority trial. *Annals of the Rheumatic Diseases.* 2019;78(6):837-843.
10. Papadopoulos DV, Koulouvaris P, Aggelidakis G, Tsantes AG, Mavrodontidis A, Papadopoulos G. Electroacupuncture for the treatment of supraspinatus calcific tendonitis. *J Clin Orthop Trauma.* 2019;10(3):624-628.
11. Pieber K, Grim-Stieger M, Kainberger F, et al. Long-Term Course of Shoulders After Ultrasound Therapy for Calcific Tendinitis: Results of the 10-Year Follow-Up of a Randomized Controlled Trial. *Am J Phys Med Rehabil.* 2018;97(9):651-658.
12. De Boer FA, Mocking F, Nelissen EM, Van Kampen PM, Huijsmans PE. Ultrasound guided Needling vs Radial Shockwave Therapy in calcific tendinitis of the shoulder: A prospective randomized trial. *J Orthop.* 2017;14(4):466-469.
13. de Witte PB, Kolk A, Overes F, Nelissen R, Reijnierse M. Rotator Cuff Calcific Tendinitis: Ultrasound-Guided Needling and Lavage Versus Subacromial Corticosteroids: Five-Year

Outcomes of a Randomized Controlled Trial. *Am J Sports Med.* 2017;45(14):3305-3314.
14. Battaglia M, Guaraldi F, Gori D, Castiello E, Arvat E, Sudanese A. Efficacy of triamcinolone acetate and methylprednisolone acetonide for intrabursal injection after ultrasound-guided percutaneous treatment in painful shoulder calcific tendonitis: a randomized controlled trial. *Acta Radiol.* 2017;58(8):964-970.
15. Clement ND, Watts AC, Phillips C, McBirnie JM. Short-Term Outcome After Arthroscopic Bursectomy Debridement of Rotator Cuff Calcific Tendonopathy With and Without Subacromial Decompression: A Prospective Randomized Controlled Trial. *Arthroscopy: The Journal of Arthroscopic & Related Surgery.* 2015;31(9):1680-1687.
16. Kim YS, Lee HJ, Kim YV, Kong CG. Which method is more effective in treatment of calcific tendinitis in the shoulder? Prospective randomized comparison between ultrasound-guided needling and extracorporeal shock wave therapy. *J Shoulder Elbow Surg.* 2014;23(11):1640-1646.
17. Sabeti M, Schmidt M, Ziai P, Graf A, Nemecek E, Schueller-Weidekamm C. The intraoperative use of ultrasound facilitates significantly the arthroscopic debridement of calcific rotator cuff tendinitis. *Arch Orthop Trauma Surg.* 2014;134(5):651-656.
18. Kolk A, Yang KGA, Tamminga R, van der Hoeven H. Radial extracorporeal shock-wave therapy in patients with chronic rotator cuff tendinitis: a prospective randomised double-blind placebo-controlled multicentre trial. *Bone Joint J.* 2013;95-B(11):1521-1526.
19. de Witte PB, Selten JW, Navas A, et al. Calcific tendinitis of the rotator cuff: a randomized controlled trial of ultrasound-guided needling and lavage versus subacromial corticosteroids. *Am J Sports Med.* 2013;41(7):1665-1673.
20. Ioppolo F, Tattoli M, Di Sante L, et al. Extracorporeal shock-wave therapy for supraspinatus calcifying tendinitis: a randomized clinical trial comparing two different energy levels. *Phys Ther.* 2012;92(11):1376-1385.
21. Tornese D, Mattei E, Bandi M, Zerbi A, Quaglia A, Melegati G. Arm position during extracorporeal shock wave therapy for calcifying tendinitis of the shoulder: a randomized study. *Clin Rehabil.* 2011;25(8):731-739.
22. Zhu J, Jiang Y, Hu Y, Xing C, Hu B. Evaluating the long-term effect of ultrasound-guided needle puncture without aspiration on calcifying supraspinatus tendinitis. *Adv Ther.* 2008;25(11):1229-1234.
23. Hsu CJ, Wang DY, Tseng KF, Fong YC, Hsu HC, Jim YF. Extracorporeal shock wave therapy for calcifying tendinitis of the shoulder. *J Shoulder Elbow Surg.* 2008;17(1):55-59.
24. Albert JD, Meadeb J, Guggenbuhl P, et al. High-energy extracorporeal shock-wave therapy for calcifying tendinitis of the rotator cuff: a randomised trial. *J Bone Joint Surg Br.* 2007;89(3):335-341.
25. Sabeti M, Dorotka R, Goll A, Gruber M, Schatz KD. A comparison of two different treatments with navigated extracorporeal shock-wave therapy for calcifying tendinitis – a randomized controlled trial. *Wiener klinische Wochenschrift.* 2007;119(3-4):124-128.
26. Cacchio A, Paoloni M, Barile A, et al. Effectiveness of radial shock-wave therapy for calcific tendinitis of the shoulder: single-blind, randomized clinical study. *Phys Ther.* 2006;86(5):672-682.
27. Sabeti-Aschraf M, Dorotka R, Goll A, Trieb K. Extracorporeal shock wave therapy in the treatment of calcific tendinitis of the rotator cuff. *Am J Sports Med.* 2005;33(9):1365-1368.

28. Krasny C, Enenkel M, Aigner N, Wlk M, Landsiedl F. Ultrasound-guided needling combined with shock-wave therapy for the treatment of calcifying tendonitis of the shoulder. *J Bone Joint Surg Br.* 2005;87(4):501-507.

29. Pleiner J, Crevenna R, Langenberger H, et al. Extracorporeal shockwave treatment is effective in calcific tendonitis of the shoulder. A randomized controlled trial. *Wiener Klinische Wochenschrift.* 2004;116(15-16):536-541.

30. Gerdesmeyer L, Wagenpfeil S, Haake M, et al. Extracorporeal shock wave therapy for the treatment of chronic calcifying tendonitis of the rotator cuff: a randomized controlled trial. *JAMA.* 2003;290(19):2573-2580.

31. Pan PJ, Chou CL, Chiou HJ, Ma HL, Lee HC, Chan RC. Extracorporeal shock wave therapy for chronic calcific tendinitis of the shoulders: a functional and sonographic study. *Arch Phys Med Rehabil.* 2003;84(7):988-993.

32. Cosentino R, De Stefano R, Selvi E, et al. Extracorporeal shock wave therapy for chronic calcific tendinitis of the shoulder: single blind study. *Annals of the Rheumatic Diseases.* 2003;62(3):248-250.

33. Perlick L, Luring C, Bathis H, Perlick C, Kraft C, Diedrich O. Efficacy of extracorporal shock-wave treatment for calcific tendinitis of the shoulder: experimental and clinical results. *J Orthop Sci.* 2003;8(6):777-783.
